# Automated MUltiscale simulation environment†

**DOI:** 10.1039/d3dd00163f

**Published:** 2023-11-07

**Authors:** Albert Sabadell-Rendón, Kamila Kaźmierczak, Santiago Morandi, Florian Euzenat, Daniel Curulla-Ferré, Núria López

**Affiliations:** a Institute of Chemical Research of Catalonia (ICIQ-CERCA), The Barcelona Institute of Science and Technology, (BIST) Av. Paisos Catalans 16 Tarragona 43007 Spain asabadell@iciq.es nlopez@iciq.es; b TotalEnergies, TotalEnergies One Tech Belgium Zone industrielle C, 7181 Feluy Belgium; c Department of Physical and Inorganic Chemistry, Universitat Rovira i Virgili Campus Sescelades, N4 Block, C. Marcel·lí Domingo 1 Tarragona 43007 Spain; d TotalEnergies Research and Technology Gonfreville, Route Industrielle, Carrefour 4, Port 4864 76700 Rogerville France

## Abstract

Multiscale techniques integrating detailed atomistic information on materials and reactions to predict the performance of heterogeneous catalytic full-scale reactors have been suggested but lack seamless implementation. The largest challenges in the multiscale modeling of reactors can be grouped into two main categories: catalytic complexity and the difference between time and length scales of chemical and transport phenomena. Here we introduce the Automated MUltiscale Simulation Environment AMUSE, a workflow that starts from Density Functional Theory (DFT) data, automates the analysis of the reaction networks through graph theory, prepares it for microkinetic modeling, and subsequently integrates the results into a standard open-source Computational Fluid Dynamics (CFD) code. We demonstrate the capabilities of AMUSE by applying it to the unimolecular iso-propanol dehydrogenation reaction and then, increasing the complexity, to the pre-commercial Pd/In_2_O_3_ catalyst employed for the CO_2_ hydrogenation to methanol. The results show that AMUSE allows the computational investigation of heterogeneous catalytic reactions in a comprehensive way, providing essential information for catalyst design from the atomistic to the reactor scale level.

## Abstract 2

AMUSE is a multiscale framework integrating detailed atomistic information on materials and reactions to predict the performance of heterogeneous catalytic full-scale reactors.

## Graphical abstract

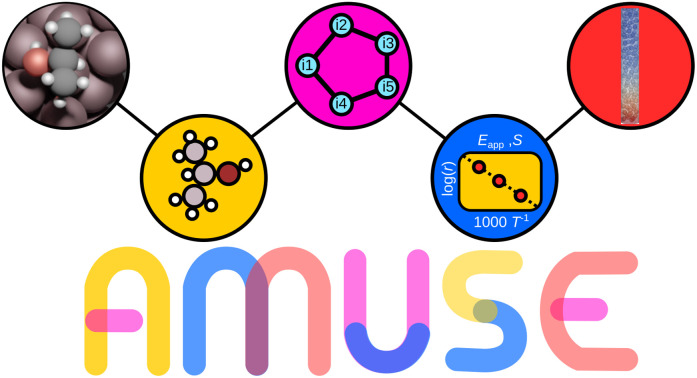

## Introduction

Modeling the performance of chemical reactors starting from first principles data has been a long-sought goal.^1,2^ Multiscale schemes that couple multiple material, length, and time scales can potentially provide essential catalytic properties such as reaction rates, conversion, and selectivity.^2,3^ However, designing reactors from *ab initio* data *via* multiscale modeling is still challenging due to two main reasons: the complexity of the catalytic reaction networks, encompassing both structure and environment; and the time scale difference between chemical and fluid dynamics phenomena.^3–7^ Ideally, a robust framework for upgrading the atomistic data to a theoretical reactor devised exclusively from first principles might be possible. Unfortunately, such computational framework does not yet exist.^3,5,7^

**Fig. 1:**
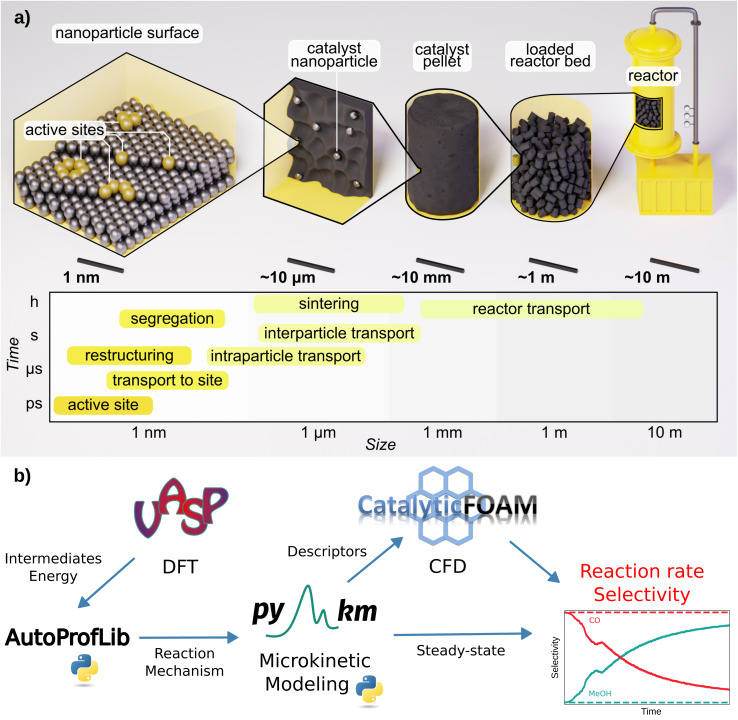
Schematics of: (a) heterogeneous catalysis and its main phenomena categorized by time and length scale, (b) AMUSE multiscale modeling workflow for heterogeneous catalysis.

The multiscale approach is illustrated in Fig. 1a. Bottom-up scheme, takes at first the information at the atomistic level of the adsorption of reaction species on the most common catalytic surfaces,^3,8^ evaluated with Density Functional Theory (DFT) simulations. Then, the connection between the different intermediates, through transition states is evaluated, and linear energy profiles are generated.^8^ Further, transition state theory is employed to determine the kinetic coefficients for the elementary reactions taking place on the surface, while the adsorption/desorption kinetic coefficients are estimated *via* the Knudsen equation.^1^ These coefficients, together with the reaction network, define the input for microkinetic models, with which the steady-state population of the surface intermediates and the rates of the elementary reactions are obtained by solving the corresponding ordinary differential equations at given experimental conditions of temperature, pressure, and reactant concentrations.^2,3,9^ Most of the studies in the field of heterogeneous catalysis end up at this level.^3,4,6^

However, to reach an adequate reactor description, transport phenomena, namely mass, energy, and momentum need to be coupled with the species balances.^5,10–13^ At the most fundamental level, these balances are defined by the Navier–Stokes and the convection–diffusion-reaction equations.^14^ The coupled equations are solved numerically with Computational Fluid Dynamics (CFD) method.^5,10–12^ In contrast, the engineering (top-down) approach employs CFD with a very simplified representation of chemical events, thus the granularity of the atomistic scale is lost. The elementary steps are compressed to just a few and the kinetic terms are fitted to experiments.^10–12,15,16^ This reduces the computational cost of the chemical steps in the CFD simulations but limits their interpretability and applicability for performance prediction. As computational power increases and numerical methods improve, the deployment of multiscale modeling in a seamless workflow becomes more achievable. Atomistic data are becoming more accessible from catalysis databases^17–21^ and robust (error estimations are now quantified),^4,22^ allowing direct experimental benchmark.^23^

Nowadays automatizing the DFT network search is mostly explored in homogeneous catalysis *via* graph theory,^24–27^ and the networks obtained are then integrated into microkinetic frameworks.^27–29^ In heterogeneous catalysis the studies have been focused on automating the search for reaction networks,^30–33^ or, separately, on developing microkinetic analysis. Microkinetic modeling for heterogeneous catalysis offers a diverse software landscape,^34^ lacking a one-size-fits-all solution in terms of versatility, source availability and cross-platform compatibility. Python-based packages CatMAP^35^ and Micki^36^ are open-source and allow the inclusion of lateral interactions. CatMAP follows a descriptor-based strategy and can model electrochemical reactions, while Micki allows the description of mass transport limitations in adsorption steps. MKMCXX^37^ is a versatile free closed-source C++ sotware for both thermo and electrocatalytic purposes, while MATLAB-based CATKINAS^38^ and Fortran-based Surface CHEMKIN^39^ are proprietary and focus exclusively on thermal applications. CERRES^40^ and DETCHEM^41^ represent additional but proprietary alternatives, being focused on reactor engineering and thermal catalysis. Coupling microkinetic modeling and automatic reaction mechanism creation was done in Reaction Mechanism Generator (RMG),^42^ which uses an experimental/computational database with heuristic-based mechanisms and applies a differential batch reactor for microkinetic analysis. In terms of including transport phenomena, the pioneering integration of a first-principles Kinetic Monte Carlo (KMC) code with CFD was achieved, and the obtained turnover frequencies were in good agreement with experiments.^43^ However, this approach implies a high computational burden. More recently, the same group devised machine learning techniques^44^ to decrease the computational cost, but they required a specific adaptation for new reactions.

In this work, we present AMUSE (Automated MUltiscale Simulation Environment), a fully automated workflow that encompasses DFT reaction data, microkinetic analysis, and CFD simulations, following the scheme presented in Fig. 1b. AMUSE was applied to the iso-propanol dehydrogenation and CO_2_ hydrogenation reactions for which DFT data was available.^45–47^ Iso-propanol dehydrogenation is a simple unimolecular reaction able to produce hydrogen^47^ while CO_2_ hydrogenation encompasses a complex network and provides a route to green methanol production.^45,46^ For both of the reactions, the simulation results were compared with the experimental data available in the literature to verify the performance of our workflow.^45–47^ A complete example of the workflow applied on iso-propanol dehydrogenation can be found in the GitHub repository (https://github.com/LopezGroup-ICIQ/amuse/tree/main/tutorial/tutorial).

## Developing an integrated methodology for multiscale modeling in heterogeneous catalysis

We have developed and tested an automated workflow able to integrate DFT data into reactor modeling through microkinetics and CFD simulations, the Automated MUltiscale Simulation Environment, AMUSE, Fig. 1b. First, the DFT reaction energy profiles are generated, and in parallel, the mechanisms are automatically identified. These two represent the ingredients for performing the microkinetic analysis, whose results are then input into the open-source CFD code. To address these tasks, we have developed two Python libraries, AutoProfLib for the reaction mechanism identification and PyMKM^9^ to build microkinetic models.

### AutoProfLib

AutoProfLib is a Python library developed in our team that processes DFT results in heterocatalytic reaction(s), to generate automatically reaction networks and obtain reaction energies. The inputs of the program are the geometries and energies of optimized structures. Currently, it is adjusted to the results obtained with VASP software, hence it requires CONTCAR and OUTCAR files. Yet, it can also take the structures as .xyz files from external databases, such as ioChem-BD.^17^

The library consists of two classes: PreProcessor and AutoProfLib. The PreProcessor class extracts automatically the coordinates of the intermediates, the gas phase molecules, and the transition states, together with their corresponding energies from the DFT output files. If frequency calculations are available, it is also possible to estimate the Gibbs and Helmholtz free energies (described in detail in Note S1.1-2 and Fig. S1;† this functionality is based on ASE^48^ Python library). Since the vibrational contribution to the entropy generates numerical discrepancies for low frequencies, AutoProfLib includes a method to either remove, replace, or treat such frequencies under a certain value according to Grimme's approach,^49^ both the specific threshold and the processing method upon user definition.

**Fig. 2:**
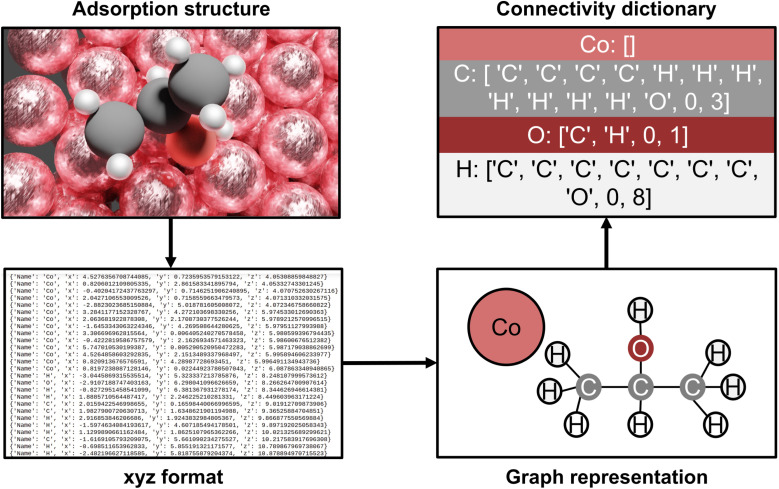
AutoProfLib workflow scheme. First, the optimized adsorption structures are converted to .xyz format. Next, the library encodes the geometric information into a molecular graph. The graph information is translated into a connectivity dictionary, which allows the comparison between all the surface intermediates to obtain the mechanism.

The AutoProfLib class transforms 3-dimensional structures of reaction intermediates and transition states into molecular graphs using the NetworkX Python library,^50^ and subsequently compares them to create the reaction network. As illustrated in Fig. 2, the structure file is parsed by the library to extract the (chemical) element types and their positions. Further, the environment of each atom in the molecule is analyzed, to establish which of them are connected, *i.e.* present within a certain bond radius (see Fig. S2†). The final output of this process is the adjacency matrix of the molecule, which is used for generating molecular graphs. Further, all the information contained in these graphs is condensed into connectivity dictionaries (see Note S.1.3†).

The link between species (*e.g.*, *i* and *j*) is established by comparing their corresponding connectivity dictionaries (*e.g.*, *n*_*i*_ and *n*_*j*_), as illustrated in Fig. S3.† To this end, a set of possible, chemically allowed, transformations was defined, including atom addition (like hydrogenation, halogenation, oxidation, adsorption of gas species) and elimination (*via* bond breaking or desorption). If after such operation two connectivity dictionaries are the same (*e.g. n*_*i*_ = *n*_*j*_), the two species are directly connected within the reaction mechanism. The transition states are assigned within the reaction network by comparing their connectivity dictionaries (*e.g. n*_TS_) with the connectivity dictionaries of the already connected reaction intermediates (*e.g. n*_*i*_*vs. n*_TS_*vs. n*_*j*_). As such the reaction network is created, and the different paths in the mechanism are identified by following all routes that link reactants (first node) and products (last node). Then, each intermediate and transition state in each path is associated with the corresponding energy, generating the energy profiles of the system.

Finally, the outputs from AutoProfLib library are the reaction network and the energy profiles. The reaction network is expressed in two different ways, the reaction network graph to visualize the interconnection between each one of the intermediates and the reaction mechanism to be used directly with the PyMKM Python library. Then, the energy profiles are also exported in two different manners, the figure of the energies of the intermediates and the transition states as a function of the reaction coordinate, and the energy inputs for the microkinetic analysis using PyMKM. Further details on the AutoProfLib can be found in Note S2, in Fig. S4,† and in the GitHub repository.

### PyMKM

PyMKM is a Python microkinetic solver for heterogeneous catalysis^9^ starting from atomistic DFT data. It allows the simulation of lab-scale catalytic reactors, systems whose main target is the evaluation of the catalyst performance in a carefully controlled environment, where the impact of the transport limitations is reduced to the minimum. PyMKM employs the differential reactor (zero conversion model) and the dynamic continuous stirred tank reactor (CSTR) as reactor models. In this study, only the differential reactor was considered. PyMKM additionally implements functionalities for studying electrocatalytic systems. Taking the reaction mechanism and related energetic profile provided by AutoProfLib and the reaction conditions (T, P, reactant concentrations) as input, PyMKM automatically constructs the species balances defining the system of coupled ordinary differential equations (ODEs). The steady-state solution of the ODE system provides the population (*i.e.*, surface coverage) of the adsorbed intermediates and the reaction rate of the elementary steps. With the differential reactor model, the rates of the adsorption/desorption reactions of the reactants/products are employed to derive experimentally accessible catalyst performance indicators, such as apparent activation energies, reaction orders, and product selectivity. PyMKM additionally provides functionalities to retrieve reaction descriptors such as the degree of rate and selectivity control (DRC and DSC)^51^ and reversibility, essential quantities for the identification of the rate-determining steps (see Fig. S4† for further details).

**Fig. 3:**
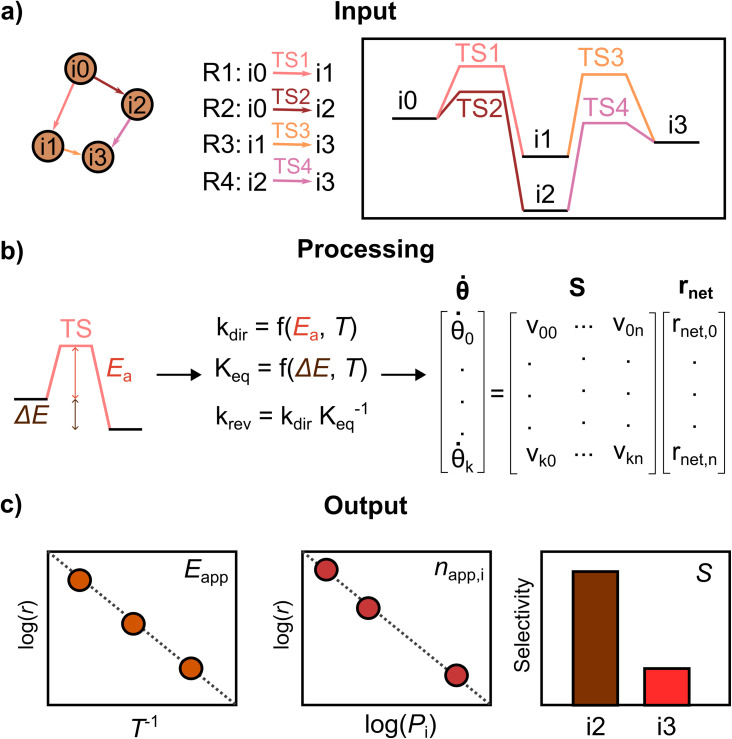
Schematic representation of PyMKM. Taking as input the mechanism and the reaction energies, translated from the reaction energy profile, PyMKM subsequently: (a) recovers the stoichiometric matrix **S** and calculates the kinetic constants for each elementary step and the net rate, ***r***_net_, (b) solves automatically the resulting ordinary differential equations system, being ***θ̇*** the derivative in time of the surface coverage vector *θ*, and (c) outputs the rates, selectivity, apparent activation energy and reaction orders.

The procedure followed by PyMKM is illustrated in Fig. 3. The stoichiometric matrix **S** is equivalent to the mechanism graph as **S** can be built using the edges of the mechanism graph as columns (*n* elementary steps) and its nodes as rows (*k* species or states). The nearest and furthest nodes from the beginning of the graph are assigned *ν*_*k*,*n*_ = −1 and *ν*_*k*,*n*_ = +1 values, respectively, for each edge.

Then it is possible to generate the coverage vector, ***θ***, from the file containing the concentration for all the *k* species on the surfaces or states involved in the mechanism. After that, the ***r***_net_ vector can be estimated following these points: (i) calculate the kinetic coefficients of the forward elementary steps according to the Arrhenius equation for surface reactions and the Hertz–Knudsen equation for adsorption/desorption processes, eqn S7 and S8,† using the reaction energy profile translated automatically by AutoProfLib into the energy input file. (ii) Estimate the kinetic coefficient of the reverse elementary steps by dividing the forward kinetic constant by the step equilibrium constant (*K* = *k*_dir_/*k*_rev_) to ensure thermodynamic consistency at the elementary level.^52^ (iii) Obtain the net reaction rate equation *r*_net_ for each elementary step *i*, being *r*_net,*i*_ = *k*_dir,*i*_*θ*_*k*_ − *k*_rev,*i*_*θ*_*k*−1_. *k*_dir_ and *k*_rev_ are the forward and reverse kinetic constants respectively.

With this, it is possible to generate the species balance for the surface intermediates, where the derivative with respect to time of the surface coverage vector ***θ̇***_*k*_ is obtained by multiplying the stoichiometric matrix by the net reaction rates vector ***r***_net_, as shown in Fig. 3b. The ***θ̇***_*k*_ vector represents a stiff coupled ODE system, which is numerically solved until steady-state in PyMKM relying on the implicit LSODA solver available on SciPy,^53^ using a double float precision, and tolerance of 10^−6^ s^−1^. The details about the input/output processing of PyMKM can be found in Note S2, Fig. S4, and the GitHub project.

### CatalyticFOAM

The outputs of the AutoProfLib and the PyMKM are used as input to CFD simulations conducted using OpenFOAM.^54^

The standard solvers in OpenFOAM have been extended and coupled with catalyticFOAM, an external tool for solving catalytic heterogeneous reacting flows with detailed kinetic mechanisms.^11^ This solver adopts an operator-splitting technique for dividing the transport and reaction problems during which a semi-batch reactor mass balance is solved where the mass exchanged by each individual species at the catalytic surface is estimated independently in each cell according to local properties and the detailed kinetics. The catalyst was localized on the surface of the spheres, and thus, diffusion in a porous media was not considered. The difference in the number of cells was primarily due to the refinement around the sphere surface. The reactor mesh was generated using the SALOME software^55^ and the OpenFOAM meshing utilities (see Note S3†). A Fixed-Bed Reactor (FBR) was employed both for its relative simplicity and the possibility to compare directly to available experimental data. Further details on the CFD simulations can be found in Note S3 and Fig. S5 and S6.† It is worth mentioning that we are presenting a CFD simplified model, which allows us to demonstrate the potential of the entire workflow. Our methodology could be extrapolated to more realistic CFD simulations, since catalyticFOAM has been applied successfully in many industrially relevant systems, such as CO transport on porous media,^56^ heat and mass transport on cellular structures,^57^ and methane partial oxidation.^58^

## Results and discussion

We have taken two different cases to assess the validity and generality of the approach. The first considered system is the iso-propanol dehydrogenation for which the complete reaction network for the Co facets (0001) and (112̄0) was reported.^47^ The reaction is unimolecular and only generates a single product, thus being the simplest example to test AMUSE.

In a second example, the robustness and versatility of the developed codes are illustrated by the CO_2_ hydrogenation to methanol, a next-generation industrial reaction in green fuel synthesis.^45,46^ This system constitutes a leap in complexity both at the reaction network level, as CO_2_ hydrogenation leads to three main products (CO, CH_3_OH, and H_2_O) and *via* several pathways, and at the material level due to the number of catalytic materials investigated with the advantage that can be benchmarked to previous experimental and computational data.

### AMUSE application to iso-propanol dehydrogenation

Alcohol dehydrogenation is a route to provide high-value-added products, namely H_2_ and ketones, *e.g.* CH_3_CHOHCH_3(g)_ ⇌ CH_3_COCH_3(g)_ + H_2(g)_.^47^ Co is an attractive catalyst due to its abundance and ability to stabilize hydrogen on the surface.^47^ Experimentally the activity and selectivity of the reaction depend on the size, shape, and modifiers of the Co nanoparticles.^47^ The reaction is structure sensitive and low-coordinated metal sites, Co(112̄0), exhibit higher reactivity than closed-packed ones, Co(0001).^47,59^

We have retrieved the DFT results for ^i^PrOH (CH_3_CHOHCH_3_) dehydrogenation on the two above-mentioned Co surfaces from literature,^47^ and plugged to AMUSE. In our simulations, we have applied the experimental temperature of 418 K, and pressure of 1 atm (*i.e.* for DFT free energy profiles, microkinetic, and CFD simulations), to compare our results with experimental trends. For CFD simulations, four meshes of different densities were used to properly capture the physics of the system (from 2.9 × 10^5^ to 2.5 × 10^6^ cells, see Table S1†).

**Fig. 4:**
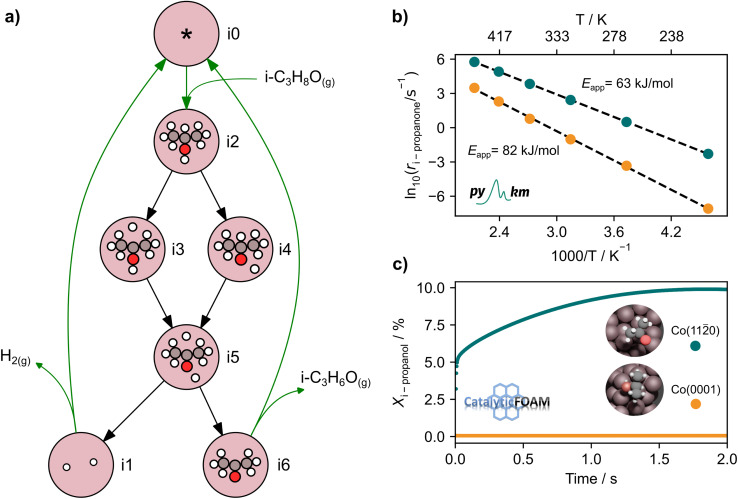
Results for iso-propanol dehydrogenation. (a) Mechanism found with the AutoProfLib for iso-propanol dehydrogenation, (b) PyMKM estimation for the apparent activation energy of iso-propanol dehydrogenation on Co(0001) and Co(112̄0), and (c) CFD-derived iso-propanol conversion trend as function of time on Co(0001) and Co(112̄0) surfaces, depicted in orange and dark-green respectively.

In the analysis with AutoProfLib, 7 different reaction intermediates were identified, including 5 adsorbed and 2 gas-phase species, and they are listed in Table 1. The mechanism is presented in Fig. 4a, and the corresponding free energy profiles are in Fig. S7 and S8.† The reaction begins with ^i^PrOH adsorption, followed by hydrogen dissociation from C or O atoms of the adsorbed CHOH group, leading to the formation of alkoxy or hydroxyalkyl intermediates, respectively. Hence two reaction mechanisms are possible. Further, subsequent CH/OH bond scission takes place, leading to the formation of iso-propanone and hydrogen, being reaction products. On Co(0001) surface, the activation energy for the O–H bond breaking of CH_3_CHOHCH_3_ is 0.59 eV, while the C–H bond breaking requires 0.93 eV. The energy difference between the initial and final states, Δ*G*, is −0.79 and 0.34 eV for O–H and C–H routes, respectively, see Fig. S7.† For the Co(112̄0) surface, the O–H and C–H bond-breaking activation energies are 0.27 and 0.61 eV, while the Δ*G* between the corresponding initial and final states are −0.84 and −0.04 eV, correspondingly, see Fig. S8.† These results have two important consequences: (i) the main route proceeds *via* alkoxy intermediate, independently of the Co surface, and (ii) the Co(112̄0) appears to be a much more active catalyst than Co(0001) since the reactions proceed faster (due to lower activation energies) and are much more irreversible (due to more negative Δ*G*).

**Table 1:** Labels for the iso-propanol dehydrogenation on Co catalyst

Intermediate	Label
*	i0
2H*	i1
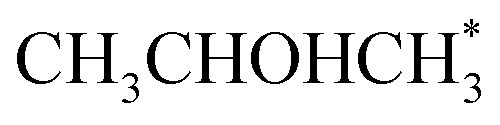	i2
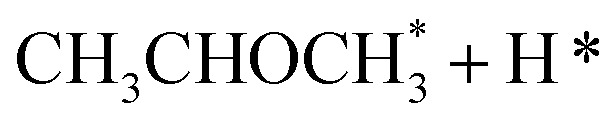	i3
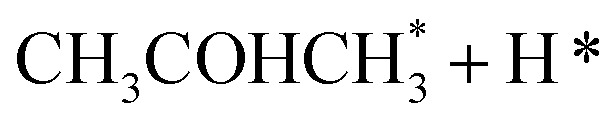	i4
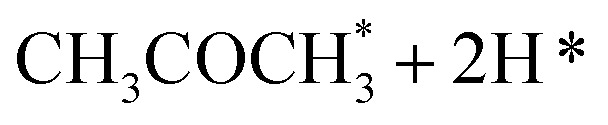	i5
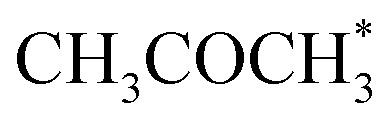	i6

The output of AutoProfLib is used as input in PyMKM, from which we obtained the apparent activation energies for the reaction. As illustrated in Fig. 4b, Co(0001) has a higher apparent activation energy than Co(112̄0), by 26 kJ mol^−1^ (0.27 eV), consistently with observations of the catalytic activity.^47^ The reaction rates are up to 6 orders of magnitude faster for Co(112̄0) at low temperatures (218 to 318 K), and up to 3 times for higher temperatures (318 to 468 K). Firstly, this difference can be attributed to the difference in stability of CH_3_CHOHCH_3_ on the two surfaces: on Co(0001) iso-propanol is physisorbed (0.03 eV), while the corresponding adsorption energy on Co(112̄0) is −0.41 eV. The activity differences can be elucidated further by the analysis of surface coverage at the steady state. The Co(0001) is fully covered by H^*^ and 
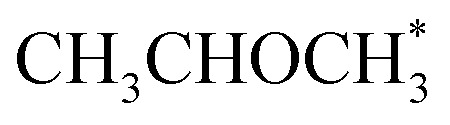
 (*θ* = 0.54 and 0.45 ML, respectively). In the case of Co(112̄0), the most abundant surface species are 
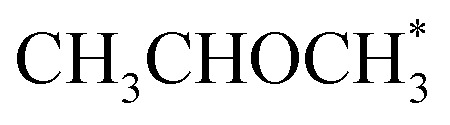
 and 
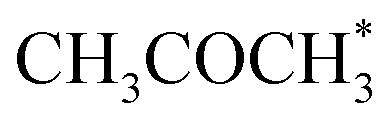
 (0.53 and 0.43 ML). Thus the reaction product is easily formed on Co(112̄0). It can be ascribed to similar thermodynamic stability between i3 and i6 on the stepped surface. Yet, due to their large stability, there is also a risk of final surface poisoning by them. On Co(0001) the product is not formed, as it is less thermodynamically stable than the proceeding intermediate, with which the surface is easily saturated.

The CFD simulations, the last step of AMUSE, reinforce our observation on activity differences, showing markedly larger activity for Co(112̄0) surface compared to Co(0001), Fig. 4c, with a grid resolution of 
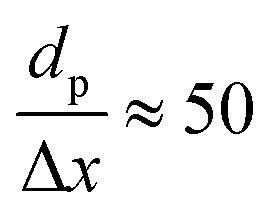
. No activity was observed for Co(0001). After reaching the steady state, the most abundant species, after the empty active site ‘*’, were i1 (H*) and i3 (
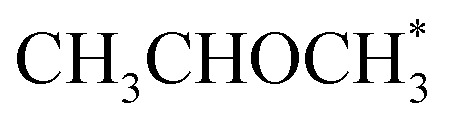
), as in the microkinetic simulations. For Co(112̄0) a 10% conversion is observed at the steady state, and the catalyst was covered with i3 intermediate (Fig. S9†). Regarding the desired product coverage, 
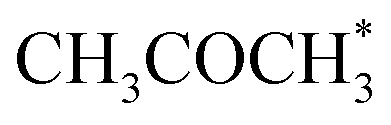
 (i6), the CFD simulations present a significantly lower value compared to microkinetic predictions since CFD simulations consider a finite system where the gas composition changes in space, contrary to the differential reactor used in our microkinetic analysis where the gas composition is constant. Thus, the equilibrium between adsorption/desorption of gaseous species is explained more accurately with CFD simulations, affecting the coverage of i6 species. These results are reinforced by the convergence test performed using different mesh resolutions and a single sphere of catalyst, see Note S3 and Table S2.†

### AMUSE application to CO_2_ hydrogenation

The next step is to study a more complex reaction network, namely CO_2_ hydrogenation (eqn (1) MeOH reaction). A new promising green route to convert CO_2_ to a liquid fuel, methanol, can be achieved *via* hydrogenation. Recently, oxide materials like indium oxide In_2_O_3_ have been proposed as catalyst^45,46^ as they inhibit the reverse water–gas shift (RWGS) reaction, (eqn (1) RWGS reaction).^45,46^ However, In_2_O_3_ is poor at activating H_2_,^45,46^ and thus small Pd contents (1–4%) are added to improve performance.^45^

The catalyst can be prepared *via* two synthetic protocols, co-precipitation (CP) and dry-impregnation (DI).^45^ Deep characterization has demonstrated that the final tridimensional structures of these synthetic routes are different. In both cases, low-nuclearity Pd clusters were responsible for the reaction but for the CP catalysts, some Pd atoms are integrated into the oxide, while in the DI case, the promoting Pd atoms sit on the surface.^45^ As a consequence, the activity and selectivity depend on the number of exposed Pd atoms.

Overall, eight different DFT catalyst models were considered as feed to AMUSE: one representing Pd(111), another one In_2_O_3_(111), while for the Pd/In_2_O_3_(111) models, for four systems Pd atoms were incorporated into the topmost layer of In_2_O_3_ lattice, representing the different nuclearities in the CP (CP_a_ to CP_d_, *N*_Pd_ = {1–4}) synthetic protocol and, in two more models, Pd atoms were added at the top of the oxide surface, referring to the DI catalysts (DI_a_ and DI_b_, *N*_exposed Pd_ = {1, 3}), see Table S3.†1
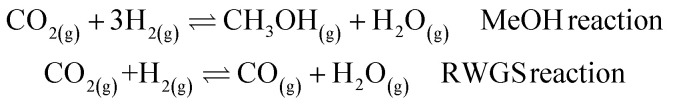


In the AMUSE simulations, the operating conditions were set according to experiments: *T* = 573 K, *P* = 5 MPa, and CO_2_ : H_2_ ratio = 1 : 4.^45,46^ A cylindrical Fixed-Bed Reactor with length *z* = 5 mm, and radius *r* = 1.25 mm was used, see Fig. S5† for visualization from CFD simulations. The CFD results convergence was tested using the same four different meshes as used for the iso-propanol case (see Table S1†).

First, the mechanism was generated using the AutoProfLib on Pd(111) and consists of 16 elementary steps involving 13 adsorbed intermediates and 5 gas phase species, Table S4 and Fig. S10.† The reaction starts with the adsorption of CO_2_ followed by hydrogenation of one of the O atoms, generating the COOH* intermediate, and then evolves into CO and water. The CO molecule can be further hydrogenated to methanol or desorb from the surface. Given the desorption energy of CO, 1.20 eV, and the barrier for the most favored CO hydrogenation pathway, 1.64 eV, reaction 7 in Table S4,† the expected selectivity towards methanol for Pd(111) at 573 K is zero.

The other composition limit, *i.e.* bare In_2_O_3_(111) has completely separated paths towards methanol and CO, Table S5 and Fig. S11† (18 elementary reactions, 14 adsorbed intermediates, and 5 gas species). If the COOH* intermediate is formed, the reaction proceeds towards the generation of CO. Otherwise, if CHOO* is produced, methanol is the major product. The reverse barrier for COOH* formation is around 0.46 eV, while the CHOO* generation is almost irreversible. Therefore, the direct analysis of the reaction energy profile suggests some selectivity towards methanol.

**Fig. 5:**
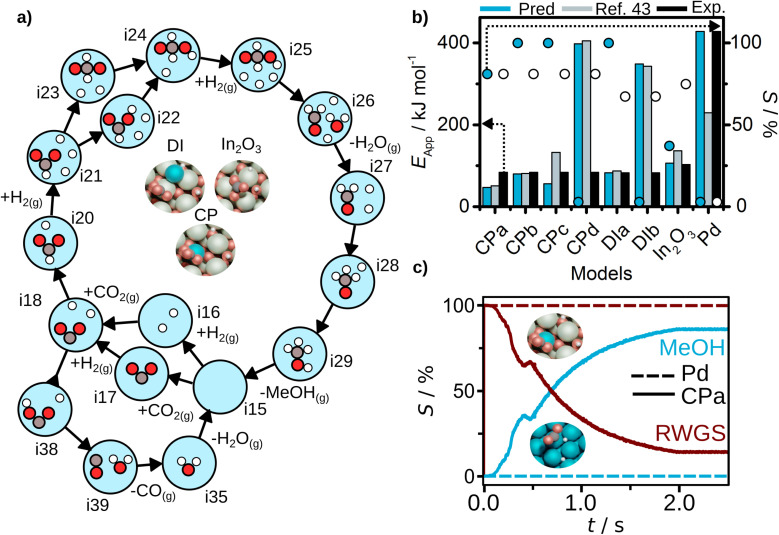
CO_2_ hydrogenation results. (a) Reaction mechanism generated for CP_a_ structure with AutoProfLib, which is general for all In_2_O_3_-based catalysts, (b) apparent activation energy (filled bars) and selectivity (dots) towards methanol formation, estimated with PyMKM for all systems, compared to experiments (black bars for apparent activation energy, white dots for selectivity) and previous computational results (blue)^45^ for all cases, and (c) CFD selectivity results for Pd(111) (dashed lines) and CP_a_ (full lines) cases.

Finally, for all the Pd-doped indium oxide cases CP_a_ to CP_d_, and DI_a_ and DI_b_, AutoProfLib identified 17 states and 5 gas species, as shown in Table S6.† The mechanism for all four CP models is shown in Fig. 5a, and involves 20 elementary steps. Compared to the In_2_O_3_ case, for the Pd/In_2_O_3_ only the CO route and the most energetically favorable pathways towards methanol are considered. The direct and reverse barriers for COOH* and CHOO* formation are listed in Table S7.† In general, methanol formation through CHOO* intermediate is favored in the models with low Pd content. Increasing the Pd content, the reaction performance converges to Pd(111). Thus, the AutoProfLib was able to accurately identify all the intermediates present in the database for all the compositions, create the reaction network, and estimate the Gibbs free energy for all the intermediates and transition states for all the materials.

Next, we estimated the apparent activation energy and the selectivity through PyMKM for all the considered systems, Fig. 5b. The outcome can be compared to the previous microkinetic model developed in MATLAB.^45^ The general trends match, Fig. 5b. The slight differences can be attributed to the change of software, MATLAB *vs.* SciPy (the later integrated into PyMKM), particularly noticeable for Pd. As the numerical value of the rates for Pd is low (10^−6^ s^−1^), errors amplify and the apparent activation energy for methanol obtained now is twice the value reported. Notably, the PyMKM results were more in line with the experiments due to a more accurate numerical approach. More importantly, our Pd(111) microkinetic model was able to explain the observed methanol selectivity, 0%, as shown in Fig. 5b. Nevertheless, we performed an additional benchmark to ensure the accuracy and usability of pyMKM using an example taken from Filot,^60^ as explained in Note S2 and shown in Fig. S12.† For the In_2_O_3_-based systems, the automated predictions and the previous computational results^45^ match remarkably well.

Among structure models for dry-impregnated catalysts, DI_a_ model corresponded the best to the experimental observations in terms of methanol apparent activation energy and selectivity, as shown in Fig. 5b, while model DI_b_ explained more appropriately the apparent activation energy for CO formation, see Fig. S13.†

Out of all the models tested, the system model that better fits the methanol apparent activation energy for the co-precipitation catalysts was the PyMKM CP_b_ with two Pd atoms, one in the lattice and the second exposed. The difference between the CP_b_ estimated and the experimental CH_3_OH activation energy is below 11 kJ mol^−1^ (0.11 eV). However, there is a discrepancy in the apparent activation energy for the reverse water–gas shift (RWGS) reaction of 125 kJ mol^−1^ (1.29 eV). In contrast, the difference of CP_a_ model for the apparent activation energy for RWGS is only 5 kJ mol^−1^ (0.05 eV). Additionally, for selectivity towards methanol, CP_a_ matches perfectly the experimental value (78% both predicted and experimental selectivity), while CP_b_ estimation deviates by 22% (100% methanol selectivity). These are remarkable results, as based on our computational predictions we can suggest that the co-precipitation catalyst likely consists of a mixture of both CP_a_ and CP_b_ structures, one more prone to generate methanol and the other one mostly responsible for CO production, and reinforces the idea that during synthesis it is not possible to fully control the population of different low-nuclearity clusters.

With PyMKM it is also possible to derive kinetic descriptors as the Degree of Rate Control (DRC) to identify rate determining steps.^51^ The DRC can be further used for the simplified analysis of reaction, significantly accelerating the performance of numerical tools (*i.e.* microkinetic and CFD simulations), or used as crucial descriptors in the correlation models with some performance properties. According to the DRC, the most relevant elementary reactions in the considered CO_2_ hydrogenation network are H_2_COOH* + 3H* to H_2_CO* + H_2_O* + 2H*, and from H_2_CO* + H_2_O* + 2H* to H_2_CO* + 2H* (R12 and R13 in Table S8, see also Note S4†). Both R12 and R13 are linked to water generation and desorption, and hence these descriptors are providing the same chemical information. Thus, for sake of simplicity, only one of the descriptors will be retained, R12. We identified CH_3_O + H to CH_3_OH, R15 as the most appropriate descriptor for methanol formation.

For cases in which reaction network become too convoluted, simplifying strategies need to be designed. To this purpose we have briefly investigated the potential of machine learning methods in accelerating the analysis of reaction kinetics and possibly identifying the source of discrepancies between predictions and experiments is demonstrated using the reaction energies of R12 and R15 to find a correlation with the apparent activation energies of the various Pd/In_2_O_3_ and In_2_O_3_ material. For that, we have used the Random Forest regressor, with a leave-one-out test method (for further details see Note S4†). We have obtained the correlation of reasonable quality, which is confirmed by the correlation coefficient (*r*^2^) and slope values of 0.98 and 0.82 for the parity plot between predicted and calculated from microkinetic apparent activation energies (Fig. S14†). Despite we are aware that external validation and a bigger data set are required to confirm the quality of the model, these results are still promising.

Finally, the CFD simulations on the best CPa model and Pd(111) systems were carried out, for which details are given in Note S3,† and the results are reported in Table S1.† For Pd(111), the conversion is null due to the difficult CO_2_ adsorption on the catalyst surface. For the considered Pd/In_2_O_3_ CPa system, a 4% conversion is retrieved from the simulations. This is remarkable as experiments give a value of about 3–4%. Also, the selectivity trends are aligned with the experimental observations (estimated selectivity towards methanol 86%, while the experimental value is 78%), as shown in Fig. 5c.

## Conclusions

We presented here AMUSE, a workflow that automates the multiscale modeling of heterogeneous catalytic reactions, taking reaction networks computed at the density functional theory level with atomistic granularity data to reactor simulations with computational fluid dynamics. The workflow has been tested to two reactions: (i) iso-propanol dehydrogenation on Co(0001) and Co(112̄0) and (ii) CO_2_ hydrogenation on Pd(111), In_2_O_3_(111), and Pd/In_2_O_3_(111) surfaces.

We began by generating the reaction mechanisms and related energy profiles for each case study using our in-house developed Python library, AutoProfLib. After that, we used the pyMKM to run the microkinetic analysis automatically. Finally, CFD simulations were performed to obtain real-life scale estimations. In all the cases, the estimations were comparable to experiments. The results presented in this work are the first instance of a general and automated workflow that goes from *ab initio* DFT data to CFD simulations. To ensure the versatility of our method, AMUSE should be applied to a wider set of materials and reactions, including carbon–carbon bond breaking, halogenations, or nitrogenations. Next, the codes will be further optimized, and the CFD simulations will be simplified using the parameters obtained with AutoProfLib and PyMKM to decrease the computational burden. The overall workflow paves the way toward multiscale integration in reactor design. Additionally, the use of AutoProfLib and PyMKM to find the optimal operating conditions (temperature, pressure, initial feed…) for CFD simulations will be also investigated.

## Data availability

AMUSE code description is available in the ESI† while the implementation and the raw DFT output data related to the iso-propanol dehydrogenation case study, following the link https://github.com/LopezGroup-ICIQ/amuse. DFT computational data related to the CO_2_ hydrogenation case study in the ioChem-BD repository following the link https://doi.org/10.19061/iochem-bd-1-106.

## Conflicts of interest

There are no conflicts to declare.

## Supplementary Material

DD-002-D3DD00163F-s001
