# Supplementary material for: Automated MUltiscale simulation environment
Source: Digit Discov. 2023 Nov 7;2(6):1721–32. doi: 10.1039/d3dd00163f (PMC10694852; doi:10.1039/d3dd00163f)
Supplement: DD-002-D3DD00163F-s001 [file DD-002-D3DD00163F-s001.pdf]

Electronic Supplementary Material (ESI) for Digital Discovery.
This journal is © The Royal Society of Chemistry 2023

Supporting Information of Automated MUltiscale Simulation Environment

Albert Sabadell-Rendón,[*,†] Kamila Kaźmierczak,[‡] Santiago Morandi,[†,¶] Florian Euzenat,[§] Daniel Curulla-Ferré,[‡] and Núria López[*,†]

†*Institute of Chemical Research of Catalonia (ICIQ-CERCA), The Barcelona Institute of Science and Technology, (BIST), Av. Paisos Catalans 16, Tarragona, 43007, Spain*

‡*TotalEnergies, TotalEnergies One Tech Belgium, Zone industrielle C, 7181 Feluy, Belgium*

¶*Department of Physical and Inorganic Chemistry, Universitat Rovira i Virgili, Campus Sescelades, N4 Block, C. Marcel·lí Domingo 1, Tarragona, 43007, Spain*

§*TotalEnergies Research and Technology, Gonfreville, Route Industrielle, Carrefour 4, Port 4864, 76700 Rogerville*

E-mail: asabadell@iciq.es; nlopez@iciq.es

Supporting Notes

Note S1: AutoProfLib details

The AutoProfLib Python library includes two classes: the `PreProcessor` class and the `AutoProfLib` class. The `PreProcessor` class, **Notes S1.1-1.2**, parses the structural and energy DFT data by reading `CONTCAR` and `OUTCAR` files from VASP simulations, and returns structures, electronic and free energy to the `AutoProfLib`. The `AutoProfLib` class, explained in Section **Note S1.3**, automatically generates the reaction mechanism, reaction energy profile, and stoichiometric matrix (the mathematical representation of the mechanism). The

mechanism and reaction energy profiles constructed by `AutoProfLib` are subsequently used by `pyMKM` Python library to automatically set the microkinetic analysis.

Note S1.1: PreProcessor class structure

The `PreProcessor` class is designed to pre-treat computational data, and its workflow is depicted in Figure S1. It has the following capabilities:

- Converts the `CONTCAR` file to an .xyz file that contains the Cartesian coordinates of the molecule (coordinates).
- Assembles the molecular structure (if requested) by processing the `CONTCAR` file (`apply_pbc`).
- Computes the Gibbs or Helmholtz free energies of the parsed systems (Gibbs, Helmholtz).

The `PreProcessor` class processes the structure (either a `CONTCAR` or a .xyz file), energies, and frequencies (from `OUTCAR`) in the user-specified directory. The user can apply the Periodic Boundary Condition for adsorbed intermediates to assemble them if required, and the coordinates of the reconstructed intermediate will be exported to the `_pbc.xyz` file. If the Gibbs method is used, the molecule's symmetry can be determined based on the .xyz file, and the frequencies are processed according to the procedure presented in **Note S1.2**. The processing of the frequencies is required to tackle the low-frequency values, which contributions artificially increase the vibrational entropy. Subsequently, regardless of the method used (Gibbs or Helmholtz), the frequencies are processed using the Erase, Substitute, or Grimme methods, based on the user's choice. The frequency list is then exported to the `freq.txt` file, and the Gibbs or Helmholtz free energies are computed. Finally, the `energy_summary.txt` file, containing the different contributions to the free energies and their corresponding values, is exported as the final summary.

PreProcessor class utilization

We provide the `UsePreProcessor.py` to use the `PreProcessor` class in the Supporting Link. The `AutoProfLib.py` file must be present in the same directory. The `UsePreProcessor.py`

script can be used to calculate the Gibbs and Helmholtz free energies separately by typing "python <PATH-TO-UsePreProcessor.py>" in the conda prompt or any other Python interpreter. The potential outputs include a .xyz file with the atomic coordinates, the frequency list (`freq.txt`), the `_pbc.xyz` file, and the `energy_summary.txt` file, containing the Gibbs or Helmholtz free energy values for the molecule or adsorbed intermediate specified by the user.

Gibbs and Helmholtz free energy calculation

The `UsePreProcessor.py` can calculate either Gibbs or Helmholtz free energies. Here its usage will be described in the example of calculating Gibbs free energies, with the explanation in the last paragraph about the differences for estimating Helmholtz free energies.

To estimate the Gibbs free energies the optimization `CONTCAR` and the `OUTCAR` files are required. They should be placed either inside the directory with the geometry optimization results or on the same level. The output will be an .xyz file with atomic coordinates, a frequency list (`freq.txt`), and an `energy_summary.txt` file containing the calculated values of Gibbs free energy for the specified molecule. To execute the script, follow these steps:

- Place `UsePreProcessor.py` and `AutoProfLib.py` in the directory with the geometry optimization results.

- In the Anaconda prompt (or any other Python interpreter), go to the desired location and type `python UsePreProcessor.py` to start the script execution (the `UsePreProcessor.py` file must be in the same folder as `AutoProfLib.py`).

- The program will ask for the type of free energy to be calculated, which in this case is Gibbs.

- Next, the program will ask for the name of the directory where the geometry optimization results are located. This directory requires `CONTCAR` and `OUTCAR` files, and the program assumes that a directory named FREQ or ../Freq, containing the `OUTCAR` from the frequency calculations, is located in the same directory or one level up.

- Afterwards, the program will ask for the initial conditions in which the Gibbs free energy will be calculated:
  - Temperature (T).
  - Molecule geometry (linear, nonlinear, or monoatomic).
  - Pressure.
  - Spin of the molecule.
  - The program will also ask how to treat frequencies of low values, with three options to choose from: Erase, Substitute, or Grimme. Under the Grimme method, the vibrational and rotational contributions are weighted according to the method proposed by Grimme et al.[1] in Reference 1, and the contributions for the lowest frequencies are penalized (tending towards 0). Additionally, the user can use the "average inertia momentum" approximation by setting max_freq[2] = True. Otherwise, the Erase and Substitute options remove or substitute the frequencies below the threshold defined by the user (in cm^{-1}, the recommended threshold is 100 cm^{-1}).

To calculate Helmholtz free energies, the launching procedure of the UsePreProcessor.py script is the same as for the Gibbs free energies. During its execution the user will be asked about the desired temperature and the way of processing the frequencies. The program will also ask if to apply Periodic Boundary Conditions to assemble the structure. If the answer will be yes, then, the program will estimate the maximum threshold (in partial coordinates) to apply the PBC (max_pbc in Inputs) based on the provided surface (typically, over 2/3rds of the surface). If the user agrees with the estimation, the PBC will be applied. Finally, the `_pbc.xyz` file will be generated in the work folder, the new coordinates will be printed as a list of dictionaries containing the atom label and the 3 spatial coordinates. After that, the Helmholtz free energy will be calculated. The frequency list and energy summary will also be saved in the path specified by the user. An example of calculating the Helmholtz free

energy can be found in the **Supporting Link**.

Note S1.2: Estimation of Gibbs and Helmholtz free energies

In this section, we will present the methodology and equations used in this work to calculate Helmholtz and Gibbs free energies, which were extracted from the documentation of the Atomic Simulation Environment (ASE) Python library.[2]

Gibbs free energy: The ideal gas approximation is commonly used to compute the Gibbs free energy (**Equation S1**). According to this approximation, particles are treated as punctual and do not interact with each other. First, the enthalpy $H(T)$ is estimated as a function of the integral of the heat capacity at constant pressure (**Equation S2**). The heat capacity C_v is the derivative of the energy as a function of temperature, and is divided into translational, rotational, and vibrational contributions, as shown in **Expression S3**. The Zero Point Energy correction is given by $E_{ZPE} = \sum_i \frac{h\nu_i}{2}$, where ν_i represents the frequencies obtained using DFT. The number of frequencies used depends on the molecule's geometry: $3N$-6 for nonlinear molecules, $3N$-5 for linear molecules, and 3 for monoatomic gases, where N represents the number of atoms in the molecule.[3]

$$G(T,P) = H(T) - S(T,P) \cdot T \tag{S1}$$

$$H(T) = E_{DFT} + E_{ZPE} + \int_0^T CpdT \tag{S2}$$

$$C_{v,translational} = \frac{3k_B}{2}$$

$$C_{v,rotational} = \begin{cases} \text{nonlinear molecule} & \frac{3k_B}{2} \\ \text{linear molecule} & k_B \\ \text{monoatomic molecule} & 0 \end{cases} \tag{S3}$$

$$\int_0^T C_{v,vibrational} dT = \sum_i \frac{h\nu_i}{e^{\frac{h\nu_i}{k_B T}} - 1}$$

PBR Next, the entropy can be estimated by applying a similar decomposition as that used for C_v, as shown in **Expression S4**. Here, P^0 represents the standard pressure (usually $1.013 \cdot 10^5$ Pa), M refers to the mass of the gas in S_{trans}, I denotes the inertia momentum in S_{rot}, and s represents the spin of the molecule in $S_{electronic}$.

$$S_{trans} = k_B \left(ln \left(\left(\frac{2\pi M k_B T}{h^2} \right)^{\frac{3}{2}} \frac{k_B T}{P^0} \right) + \frac{5}{2} \right)$$

$$S_{rotational} = \begin{cases} \text{nonlinear} & k_B \left(ln \left(\frac{\sqrt{\pi I_a I_b I_c}}{\sigma} \left(\frac{8\pi^2 k_B T}{h^2} \right)^{\frac{3}{2}} \right) + \frac{3}{2} \right) \\ \text{linear} & k_B \left(ln \left(\frac{8\pi^2 I k_B T}{\sigma h^2} \right) + 1 \right) \\ \text{monoatomic} & 0 \end{cases} \tag{S4}$$

$$S_{vibrational} = k_B \sum_i \left(\frac{h\nu_i}{k_B T \left(e^{\frac{h\nu_i}{k_B T}} - 1 \right)} - ln \left(1 - e^{\frac{-h\nu_i}{k_B T}} \right) \right)$$

$$S_{electronic} = k_B ln \left(2 \cdot s + 1 \right)$$

Helmholtz free energy: The Helmholtz free energy (**Equation S5**) is computed considering only the vibrational contributions to heat capacity and entropy, and employs the $3N$ frequencies ν. First, the internal energy U is estimated using **Equation S6**. Then, the entropy is calculated using $S_{vibrational}$ from **Expression S4**.

$$F(T) = U(T) - S(T) \cdot T \tag{S5}$$

$$U(T) = E_{DFT} + E_{ZPE} + \sum_i \frac{h\nu_i}{e^{\frac{h\nu_i}{k_B T}} - 1} \tag{S6}$$

Note S1.3: the AutoProfLib class structure

The `AutoProfLib` generates the mechanism by producing the molecular adjacency matrix and connectivity dictionaries for all the intermediates in advance. The `AutoProfLib` parses the .xyz files provided by the user or created with the `PreProccessor` class, and stores the coordinates and labels of the atoms as a list of dictionaries. Subsequently, the adsorbed molecules are identified and separated from the surface. The connectivity between the molecules is then determined using the `is_in_the_sphere` function, which operates based on the scheme shown in **Figure S2**. The is_in_the_sphere function creates a sphere centered on each atom of the molecule, and the neighboring atoms that lie within the sphere's radius are deemed to be bonded to the central atom. For each iteration (referred to as i in **Figure S2**), the radius of the sphere increases until either: a) the maximum number of bonds of the central atom is achieved, or b) the radius is greater than or equal to the maximum interatomic distance of the central atom (as specified in the `periodictable.csv` file).

Once the adjacency matrix for all the molecules parsed by `AutoProfLib` has been defined, the connectivity dictionaries are generated. As illustrated in **Figure S3**, the connectivity dictionaries contain the following keys:

- Empty: this key determines if the intermediate is the empty surface, the empty surface with a vacancy, or neither. The value of the key is 1 for the first two cases and "" for the last.
- One label for each element in the system. In the example depicted in **S3 d)**, these keys correspond to C, O, and H. The corresponding value is a list comprising the total number of bonds of all the atoms labeled as the key in the intermediate, the number of

atoms labeled as the key but not bonded to the principal chain, and the total number of atoms labeled as the key present in the intermediate.

Thus, the example connectivity dictionary can be interpreted as follows: the species consists of 3 carbon atoms bonded together, one of which is attached to 2 other carbon atoms, 1 H, and 1 O. The remaining two carbon atoms are bonded to 1 carbon and 3 H each. Moreover, there are no free oxygen or hydrogen atoms in the system, and the oxygen atom is bonded to 1 H.

Subsequently, the connectivity dictionaries are employed to establish connections between different intermediates and build the mechanism as a graph using the reactive test functions. To match the intermediates (e.g. n_i and n_j) a node comparison (i.e. reactive test) function is used. Within this function, the reactive processes should be specified, which involves adding atoms (e.g. hydrogenation, halogenation, oxidation, gas species adsorptions) and atoms separation (e.g. via bond breaking and desorptions). If any transformation on n_i results in n_j, such that $n_{i,transformed} = n_j$, then f(n_i, n_j) = 1 and n_i and n_j are connected. Otherwise, f(n_i, n_j) = 0 and n_i and n_j are not connected. The outcome of applying the function f(n_i, n_j) to all species in the reaction network is the system adjacency matrix with elements a_{ij} equal to 1 if intermediate i is connected to j and 0 otherwise.

To exemplify how the reactive test functions operate, we have used the oxygen_hydrogenation_test on the state containing adsorbed CH_3CHOCH_3+H molecules (labeled as i3) and the state containing adsorbed iso-propanol (labeled as i2), as shown in **Figure S3 a)** through **c)**. The oxygen_hydrogenation_test generates a copy of i3 and adds a hydrogen bond to the oxygen of that copy by modifying the O and H entries (**Figure S3 d)** to **e)**). Subsequently, the modified version of i3's connectivity dictionary is compared to all other intermediates. If the modified dictionary is identical to another intermediate, those two states are connected. In the example in **Figure S3**, i3 and i2 states are connected.

Then, by applying the test functions iteratively to all the intermediates, the mechanism graph is generated. Finally, utilizing this graph, the connections between intermediates are

related to the energies for each one of the intermediates and transition states, applying the reference stated by the user, and generating the reaction energy profile.

Note S2: Details on PyMKM

The inputs for the PyMKM are the reaction mechanism, the energy profile, and the operating conditions (T, P, gas phase and surface compositions) generated with the AutoProfLib, **Figure S4**.

The mechanism input is parsed using the `rm_parser.py` file, provided in the **Supporting Link**. This file, named `rm.mkm`, includes the following:

1. The first few lines represent global reactions in the gas phase, with each global reaction having the syntax: Label of the global reaction (to be set by the user) index of the main product desorption reaction: global reaction in gas phase (automatically generated).

2. Three blank lines are used to separate the global reactions from the elementary steps.

3. A list of elementary steps follows. Each elementary step has the following syntax: reactant 1 + reactant 2 $\rightarrow$ product 1 + product 2. The three last characters of the gas phase molecules should be "(g)".

The energy input file, `g.mkm`, is parsed using the `g_parser.py`. The syntax of `g.mkm` is as follows:

1. The first part of the file consists of a list of TS energies. The number of entries should be equal to the number of elementary reactions present in the mechanism file. Each entry in the TS energies list has the following syntax: internal energy (or enthalpy), blank space, entropy in electron-Volt per K at certain temperature ($eV \cdot K^{-1}$). The syntax is general for all the input energy files.

2. Three blank lines separate the TS energies list from the next section.

3. The next section is a list of energies of intermediates.

4. Three blank lines separate the energies of the intermediates list from the next section.

5. The last section is a list of energies of gas species. In the example shown in the **Supporting Link**, the gas phase values are 0 because the provided energies are referenced.

Given these inputs, the stoichiometric coefficients, barriers, and reaction energy increments for each elementary reaction in the mechanism are automatically calculated by pyMKM. These parameters are used to calculate the kinetic constants (k) via the Eyring equation (Equation S7) for surface reactions, where k_B is the Boltzmann constant, h is the Planck constant, E_a is the activation energy for the elementary step, and T is the temperature in Kelvin. For adsorption/desorption processes, the Knudsen-Hertz equation is used instead (Equation S8), where p is the partial pressure of the adsorbed gas species, A is the adsorption site area, and M is the mass of the gas species. The user can select the type of reactor to be simulated, either a differential or a dynamic Continuous Stirred Tank Reactor (CSTR), both implemented in the `reactor.py` file. For the CSTR reactor, the user can set the radius (radius) and the length (length) of the reactor, the inlet volumetric flow rate (Q), the catalyst mass (m_{cat}), the BET surface (S_{BET}), and the area of the active site (A_{site}).

$$k = \frac{k_B T}{h} e^{-\frac{E_a}{k_B T}} \tag{S7}$$

$$k = \frac{pA}{\sqrt{2\pi M k_B T}} \tag{S8}$$

After calculating the direct kinetic constants, the reverse kinetic constants can be estimated as illustrated in **Figure 3 b)** in the main text, which involves calculating the equilibrium constant K_{eq} from the exponential term of the Arrhenius equation and the free energy difference between the initial and final states instead of the barrier. These tasks are performed with the utilities contained in the `thermo.py` library, developed at ICIQ, which can also be used for thermodynamic consistency testing. Additionally, a reversibility test can be set by the user.[4]

The Ordinary Differential Equation (ODE) system is automatically generated with this information, as explained in the main text, and then solved using the single_run method. This method iteratively modifies the temperature and pressure values to calculate the apparent reaction orders and energy.

The Degree of Rate Control (DRC)[5] can also be calculated by the user using **Equation S9**, where r^+ is the direct rate and G_j^{TS} is the free energy of the studied transition state. The Degree of Selectivity Control (DSC) can also be calculated by performing slight perturbations in the intermediate and TS energy values using the single_run method. Additionally, it is also possible to estimate the apparent reaction orders of the reactants.

$$\chi_{RC,i} = \frac{1}{RT}\frac{\delta ln\left(r^+\right)}{\delta ln\left(G_j^{TS}\right)} \tag{S9}$$

To ensure the accuracy and usability of our microkinetic framework, we reproduced the example reported in the work of Filiot.[6] This example is the mechanism of the transformation of generic species A into B in a single unimolecular step, including the adsorption and desorption of reactant and product. To perform a 1-to-1 comparison with the benchmark, we adapted the pre-exponential factor of the kinetic constant used in the example, which was approximated to 10^{13} instead of $\frac{k_B T}{h}$ (being T the temperature, and k_B and h the Boltzmann and Planck constants respectively). As shown in **Figure S12**, our predicted surface population along time are identical to the ones presented in the example from 200 to 850 K.[6]

Note S3: Details on the CatalyticFoam simulations

The reactive flow at the catalyst grain scale is described by the multispecies, compressible, Navier-Stokes equations. We used CatalyticFoam to perform computational fluid dynamics (CFD) simulations in this work, based on the general multi-physics solver OpenFOAM[7–9] and solving the conservation of total mass, mixture momentum, mass fraction for each species and

mixture energy. Technically, since AMUSE is fully modular, it would be possible to combine it with any other CFD after adapting the input/output functionalities of our workflow. However, AMUSE is specifically designed to be combined with CatalyticFoam, which is an accurate, versatile, and robust tool. By means of CatalyticFoam, homogeneous gas-phase reactions can be included and a specific boundary treatment of heterogeneous catalytic walls for mass and heat balance was included to couple the microkinetic models. In addition, a splitting-operator approach is used to solve the species mass fraction and energy equations by successively solving and updating fields for reaction and convection-diffusion sub-problems within a time iteration. The catalytic surface coverage evolution of individual adsorbed species is also updated during the reaction operator. The CatalyticFOAM solver enables the treatment of reaction on the catalyst particle surface and the physical process related to reaction-diffusion within the catalyst grain is not considered in this paper. In the present study, a mesh convergence study on a single isolated sphere is performed in the flow regime of interest ($\mathrm{Re} = \frac{\rho v d_p}{\mu} \approx 5$), estimated from[10] to assess the grid resolution independence of the computed solution. Here, ρ stands for the density of the gaseous phase, v is the gas velocity, d_p is the catalyst particle diameter and μ is the gas viscosity. For the case of a single sphere, 10 different meshes were generated corresponding to grid resolution $\frac{d_p}{\Delta x}$ ranging from 5 to 100. In addition, a boundary layer composed of 5 layers with an expansion ratio of 1.15 is added close to the sphere surface to ensure that the code captures the concentration gradients. The meshes are realized using the blockMesh and snappyHexMesh utilities from the OpenFOAM software. For each case, the overall conversion $X_{CO} = 100 \cdot \frac{Q_{CO,in} - Q_{CO,out}}{Q_{CO,in}}$ is followed until steady-state, and shown in **Table S2**. From **Table S2**, a grid resolution corresponding to $\frac{d_p}{\Delta x} = 60$ is fine enough to fall below 5% error with the M9 reference case with reasonable computational time for Co and M7 is required for Pd and In_2O_3-based catalysts.

To ensure accuracy in our simulations, a mesh convergence study was also conducted over a reactor bed for both iso-propanol and the CO_2 hydrogenation systems. In order to avoid grid snapping problems of spheres with the snappyHexMesh for complex bed configurations

and following the guidelines of the resolution found for a single isolated sphere the open-source Salome meshing tools were used to generate the meshes used in this work. It was found that a resolution of $\frac{d_p}{\Delta x} \approx 50$ is dense enough to properly capture the complexity of the system, obtaining the most robust estimations. Then, for the CO_2 system, the results of the mesh convergence test are shown in Table S1, where we observed that conversion was only achieved for the denser meshes ($\frac{d_p}{\Delta x} \approx 60$). Considering the tests for both systems, the global results suggest that the branching of the chemical mechanism and the type of catalyst (either metal or oxide) also play an important role in CFD simulation computational performance (i.e. reducing mechanism complexity may drastically improve the CFD performances).

The workflow for adapting the microkinetic model to OpenFOAM is illustrated in **Figure S6**, showing that the workflow is fully automated until the kinetic input of CFD simulations. The AutoProfLib function uses the OpenFOAM_Mechanism function to convert the mechanism generated with AutoProfLib into elementary reactions, which are written in catalyticFoam syntax. Meanwhile, the energy profile is plotted and the intermediates, species in gas phase, and Transition State energies are exported in the `g.mkm` and the `g_ref.mkm` files. The `rm.mkm` and `g.mkm` (or `g_ref.mkm`) are the input files for pyMKM, which calculates the barriers (direct and forward) for each reaction in the `rm.mkm` file. Finally, the `Auto_MKM_toOF.py` script combines the AutoProfLib and pyMKM information, generating the OpenFOAM mechanism.

To run the simulation, the user needs to find the NASA polynomials in CHEMKIN syntax for each species in the system, which can be obtained from databases such as the 3rd Millennium Database.[11] The user also needs to find the transport parameters for each species, which can be found in the OpenFOAM examples database. Finally, the kinetic, thermochemical, and transport information is processed using the `CatalyticFoam_CHEMKINPreProcessor` script, which is included in CatalyticFoam.

Note S4: Key descriptors identification for CO_2 hydrogenation systems

We performed the Degree of Rate Control (DRC) analysis[5] to identify the most relevant elementary steps in a given mechanism. As an example, we report the DRC results for the CP_a model in Table S7. However, it has to be kept in mind that the DRC may differ for each In_2O_3-based model. As described in the main text, R15 reaction, H_3CO + H to H_3COH, was identified as crucial for MeOH formation, and R12, H_2COH to H_2CO + OH, was chosen to describe the concurring RWGS reaction. We then decided to try to create a correlation between the calculated apparent activation energies for the various Pd/In_2O_3 (including In_2O_3 by itself) materials, and reaction energies of R12 and R15 steps. For that, we have used Random Forest model, implemented in the Analytic nodes of KNIME open-source software. The number of used features was limited to 2, the depth of a decision tree was also limited to 2, and 150 trees were created for a random forest run. To test the model during its creation, leave-one-out method was applied. The parity plot between the predicted and calculated in microkinetic analysis apparent activation energy can be found in **Figure S14**. The correlation is of good quality, as proven by the values of correlation coefficient of 0.98, and slope of 0.82. However, due to the very small size of the dataset, it was not possible to perform an external validation of the model. Yet, this approach demonstrates that using only two energy descriptors could be enough to properly describe the material performance and accelerate the whole analysis.

Supporting Figures

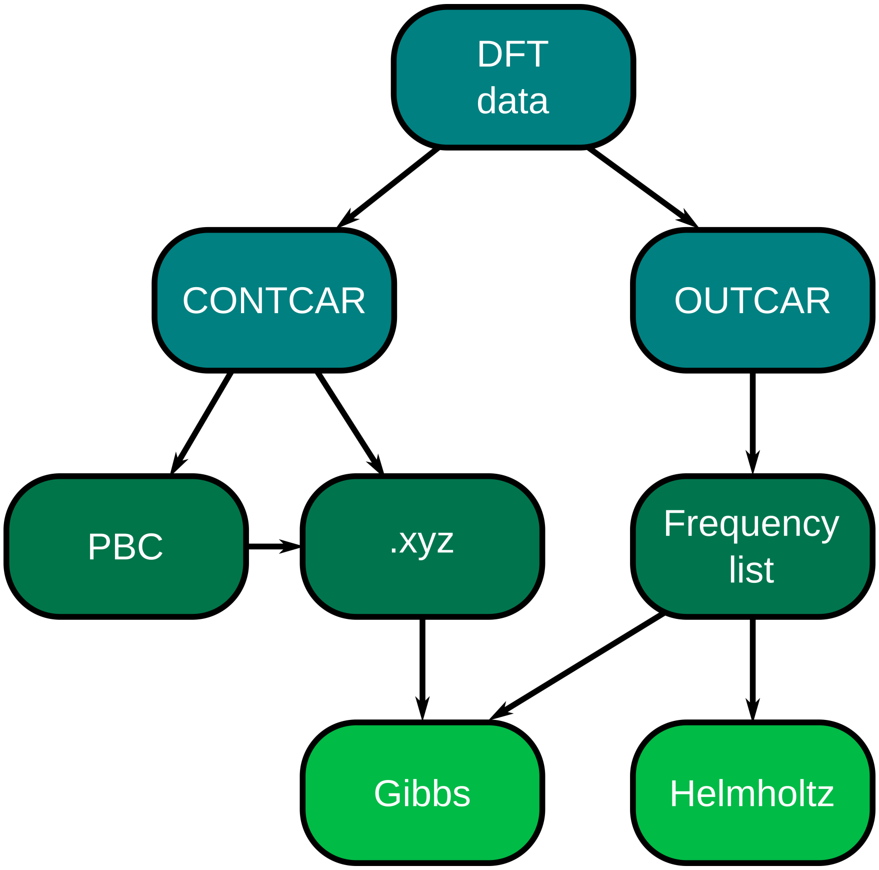

Figure S1: Workflow scheme of the PreProcessor class.

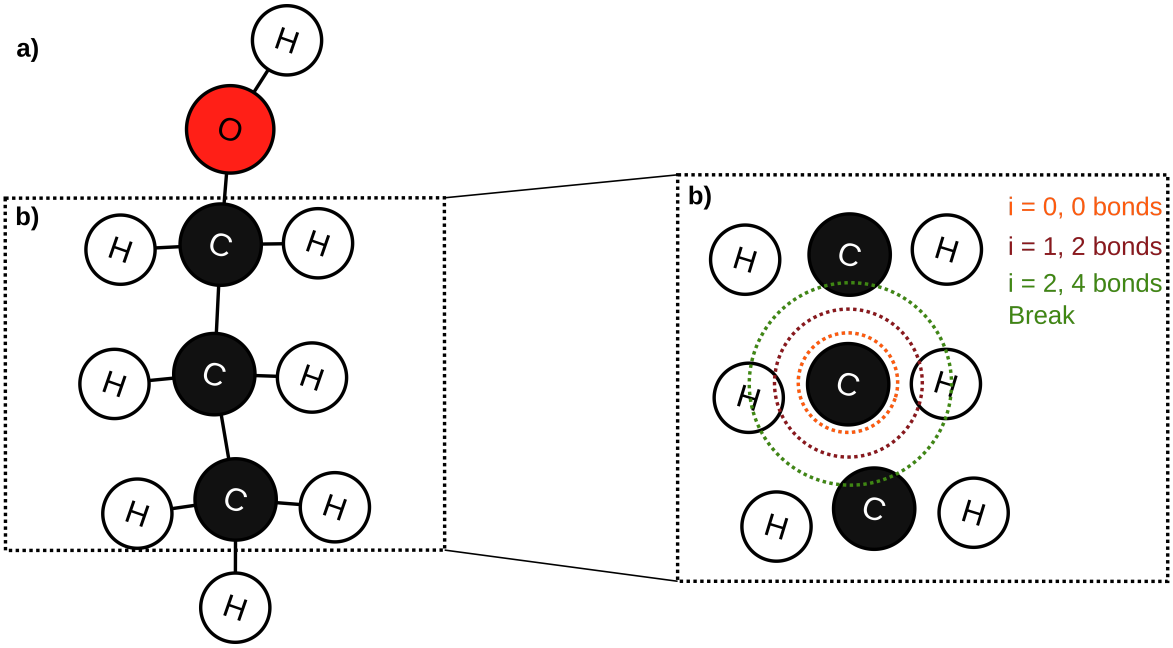

Figure S2: Scheme explaining how the `is_in_the_sphere` function works using the central carbon of 1-propanol as an example: **a)** iso-propanol graph, **b)** example of the is_in_the_sphere algorithm applied on the central atom of iso-propanol.

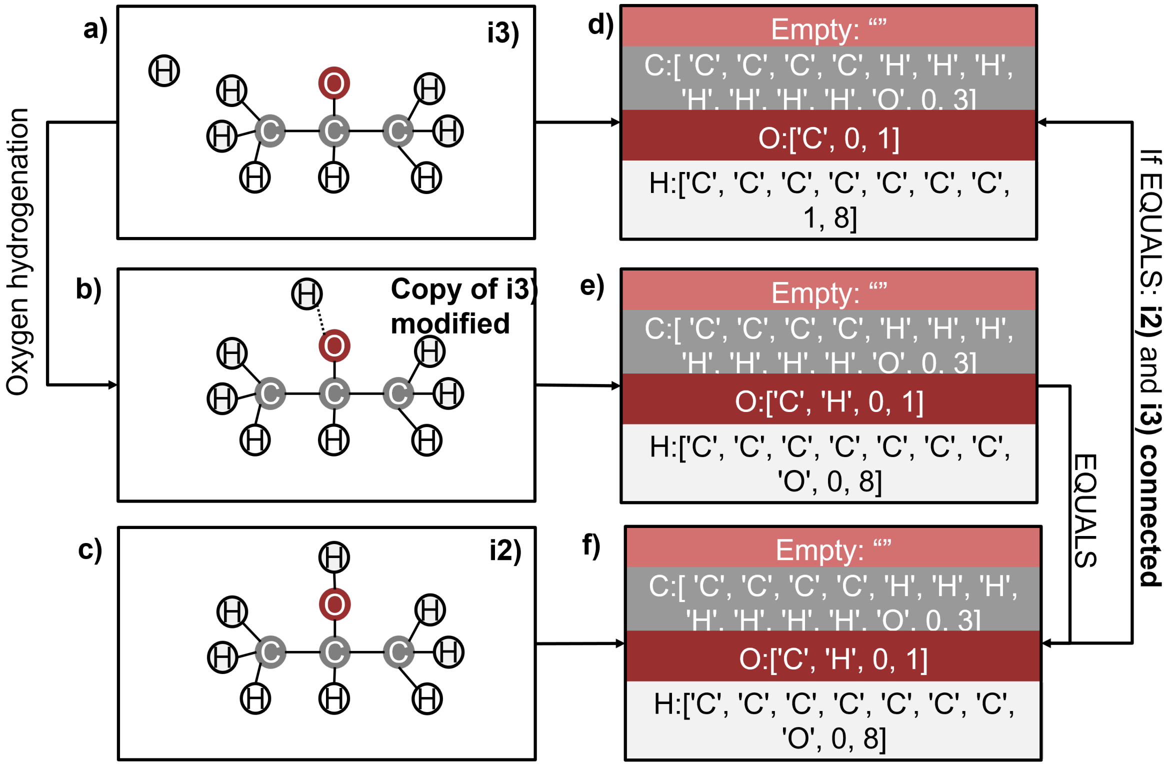

Figure S3: Example of using the connectivity dictionaries to generate the system adjacency matrix.

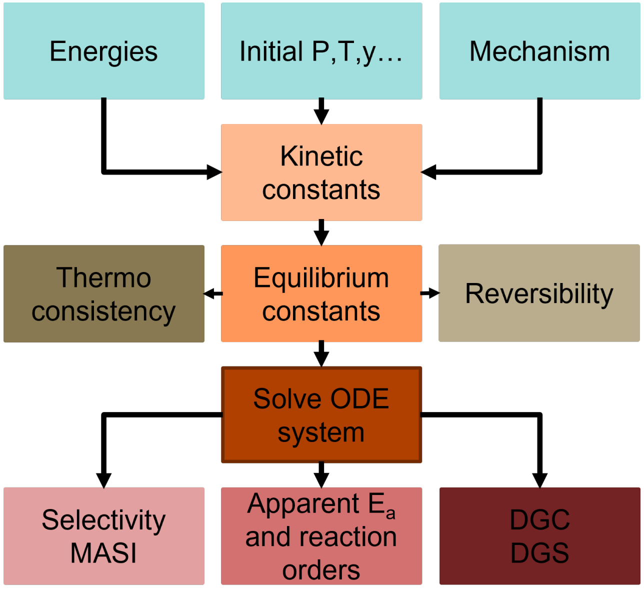

Figure S4: Scheme of the pyMKM internal workflow. MASI stands for Most Abundant Surface Intermediate.

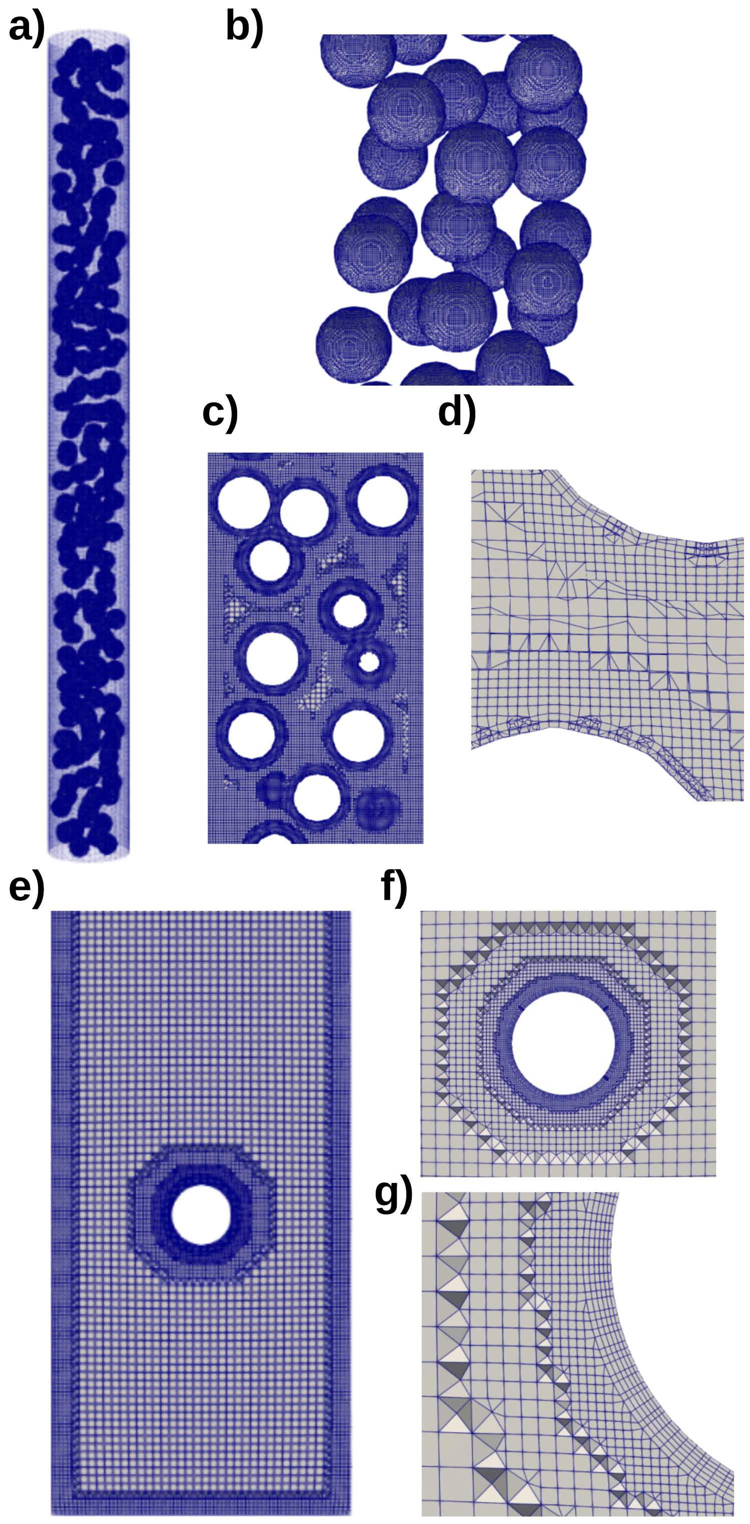

Figure S5: Schematic representation of the CFD simulation meshes: **a)** General view of the simulated Fixed-Bed Reactor, **b)** Detail on the spheres of **a)**, **c)** slice of **b)**, **d)** Detail around one of the spheres shown in **c)**, **e)** Slice of the general view of the single sphere model, **f)** Zoom around **e)**, showing the detail of the mesh, and **g)** Detail on **f)**. The single sphere model corresponds to M6 meshing, while the Fixed-Bed corresponds to M7 (see **Table S2**).

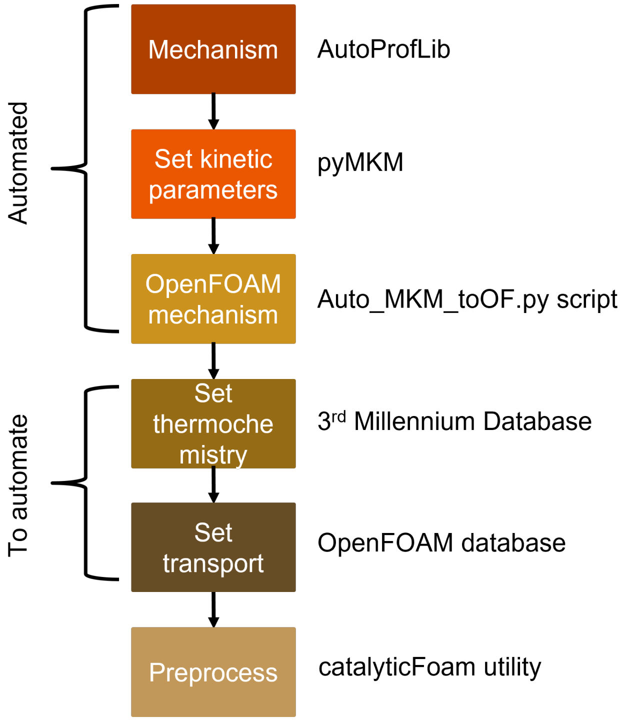

Figure S6: OpenFOAM kinetic workflow.

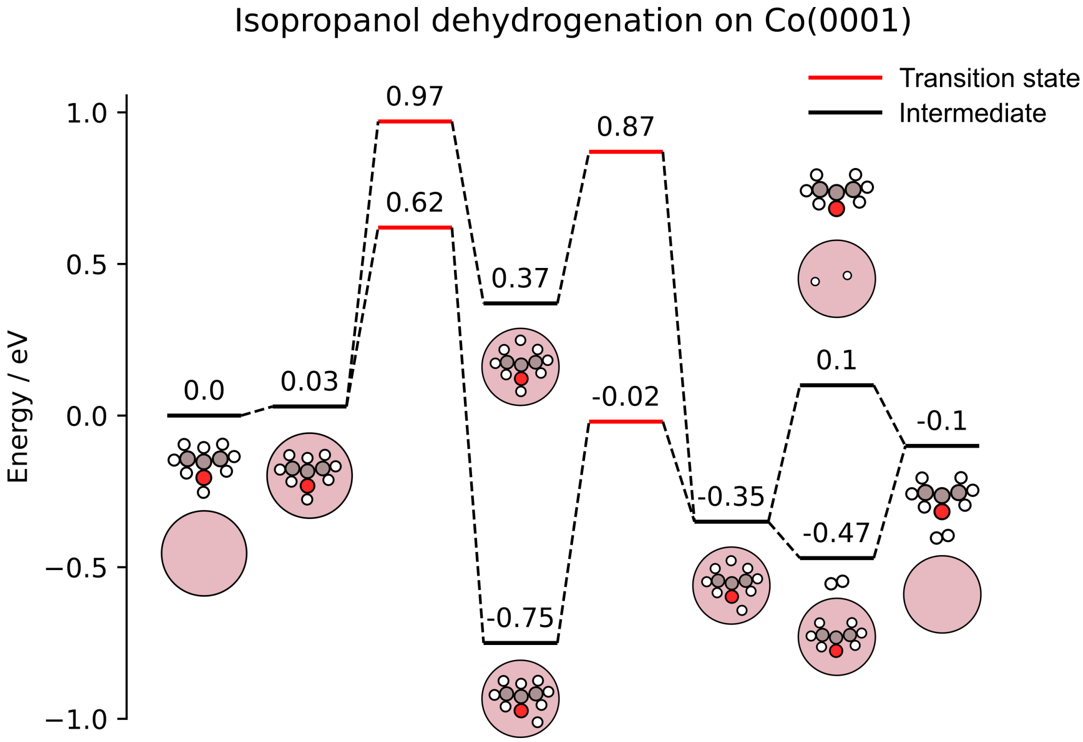

Figure S7: Energy profile for iso-propanol dehydrogenation on the Co(0001) surface.

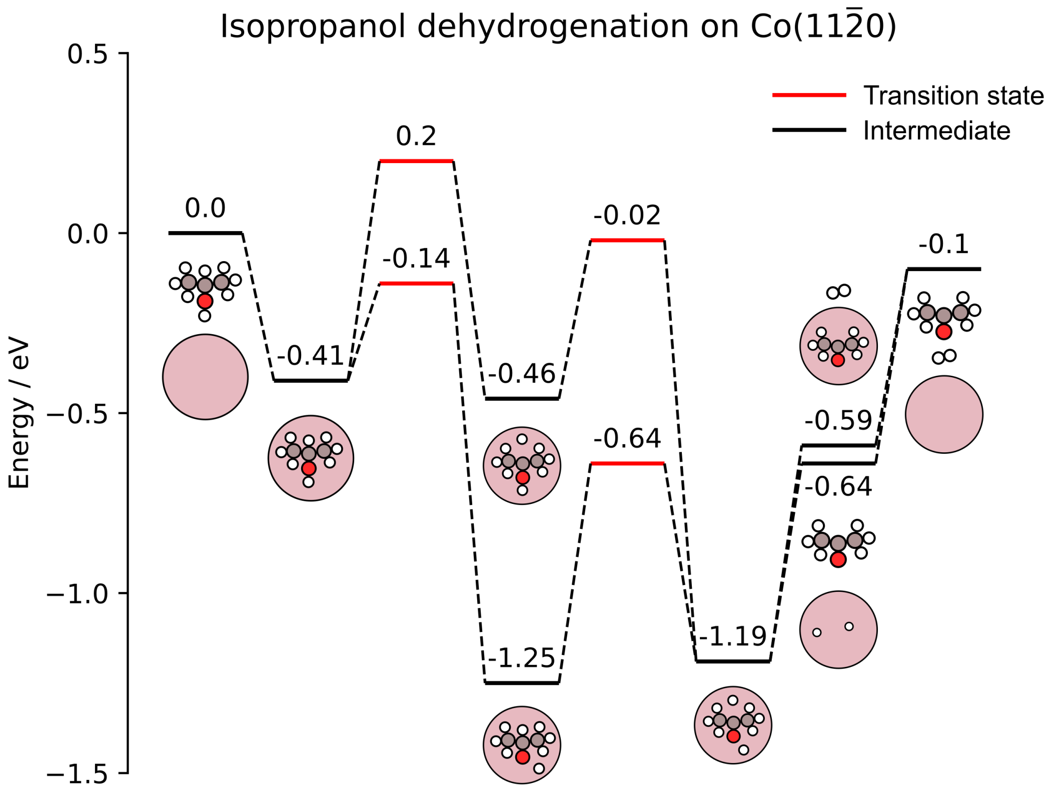

Figure S8: Energy profile for iso-propanol dehydrogenation on the Co(11$\bar{2}$0) surface.

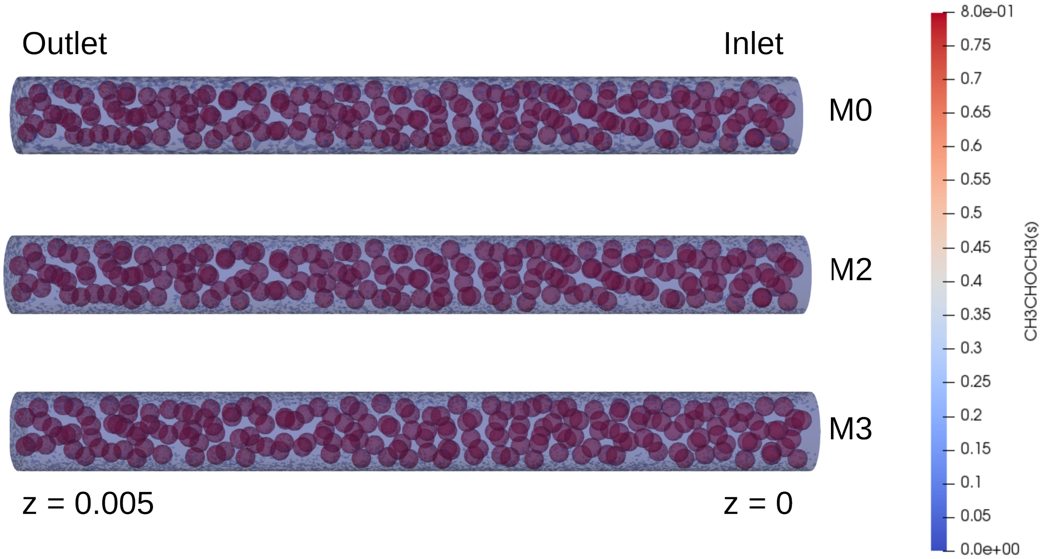

Figure S9: Population of the CH_3CHOCH_3 intermediate on the CFD simulation after 2 seconds on meshes M0, M2, and M3.

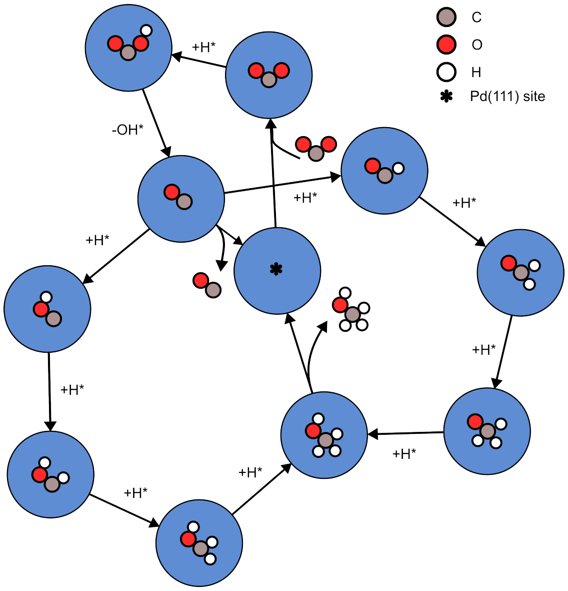

Figure S10: Reaction mechanism visualization for the CO_2 hydrogenation to methanol on Pd(111).

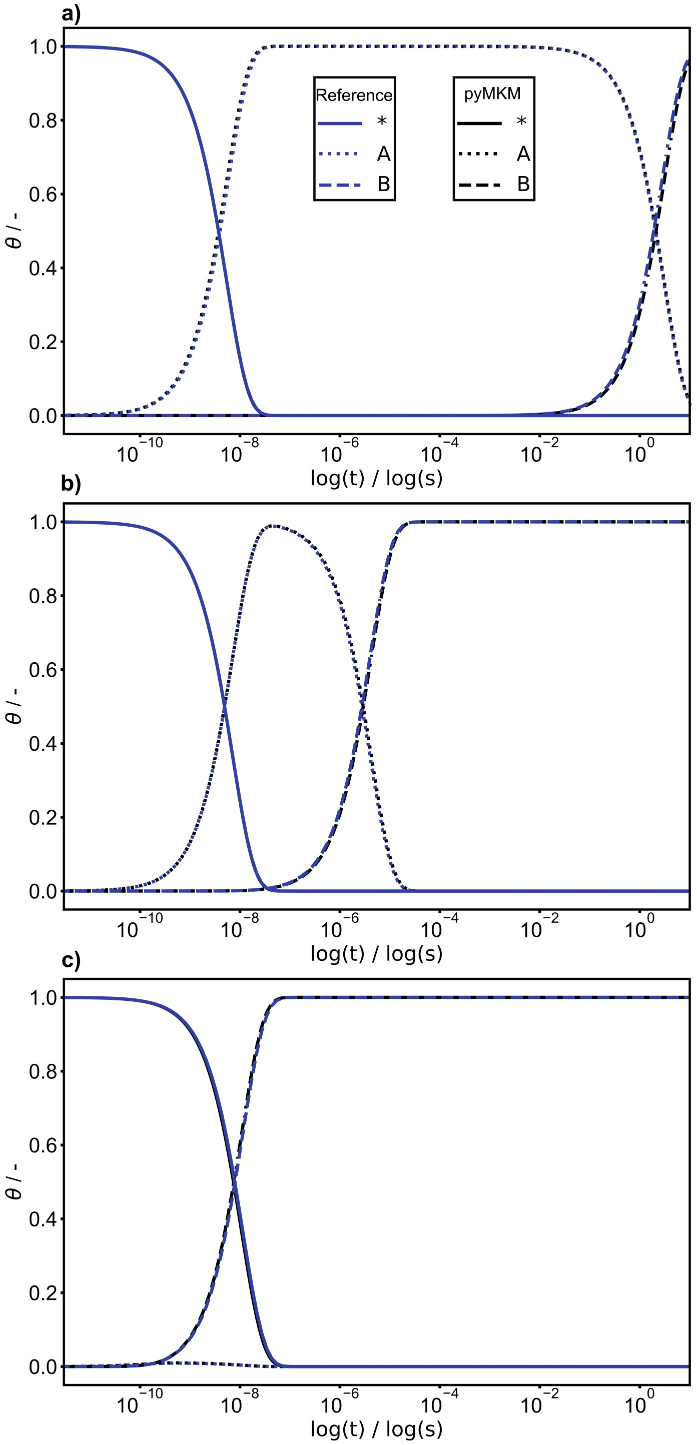

Figure S11: PyMKM species distribution benchmark for a generic species A being transformed to B, including the adsorption and desorption steps for both reactants and products at: **a)** 200 K, **b)** 350 K, and **c)** 800 K. The energies and the reference populations (in grey) were taken from reference 11,[6] and compared with our PyMKM predictions (black).

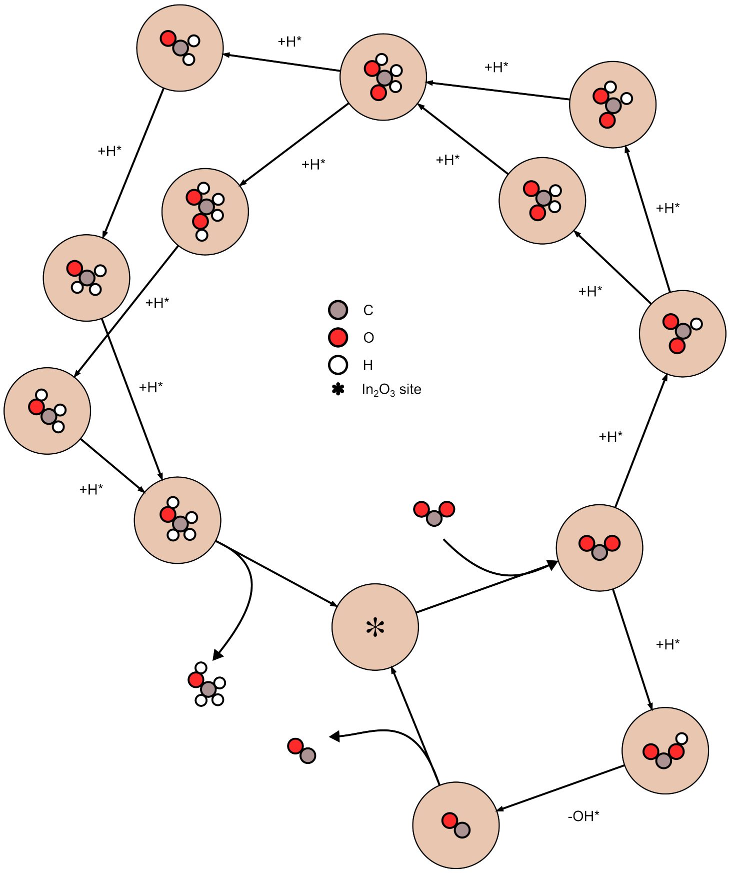

Figure S12: Reaction mechanism visualization for the CO_2 hydrogenation to methanol on the In_2O_3 surface.

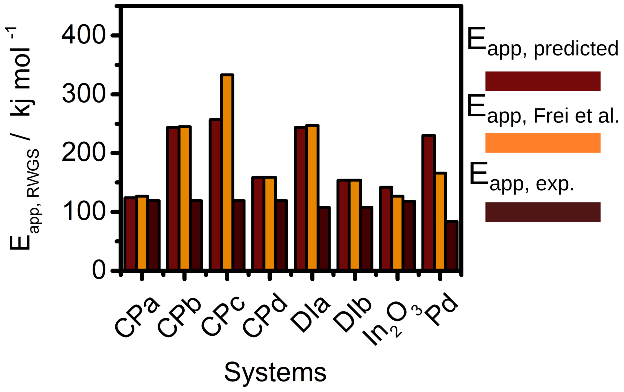

Figure S13: Apparent activation energy estimated with **PyMKM** for the CO_2 hydrogenation systems for the CO (RWGS) production for all the systems compared to previous computational results and experiments[10]

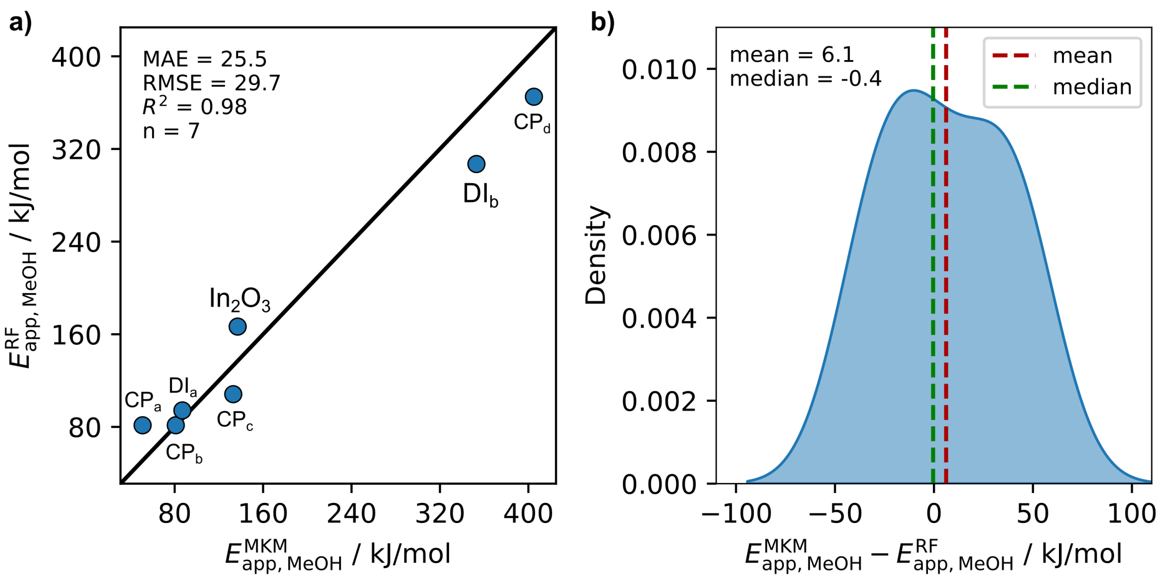

Figure S14: Random Forest model for predicting the apparent activation energy of the methanol reaction. a) Parity plot between microkinetic model (MKM) output and random forest (RF) model for the investigated materials and b) related error distribution.

Supporting Tables

Table S1: Summary of the conversion (X) results of CFD simulations for the CO_2 hydrogenation on the Pd(111) and CP_a cases and iPrOH on Co(0001) and Co(11$\bar{2}$0).

Model	Cells $\cdot 10^5$	$X_{Pd(111)}/\%$	$X_{CPa}/\%$	$X_{Co(0001)}/\%$	$X_{Co(11\bar{2}0)}/\%$
M0	2.9	0.0	0.0	0.0	10.0
M1	9.6	0.0	0.0	0.0	10.0
M2	9.7	0.0	0.0	0.0	10.0
M3	24.5	0.1	4.0	0.0	10.0

Table S2: Summary of the conversion (X) results from CFD simulations for the iPrOH dehydrogenation on the Co(11$\bar{2}$0) and CO_2 hydrogenation on the CPa model for a single sphere.

Model	Cells / d_p	$X_{Co(0001)}$/%	CPa /%
M0	5	3.68	0.09
M1	10	3.66	0.06
M2	15	3.85	0.01
M3	30	4.37	0.15
M4	50	4.73	0.46
M5	60	4.83	0.54
M6	70	4.91	0.59
M7	80	4.99	0.63
M8	90	5.05	0.67
M9	100	5.06	0.69

Table S3: Microkinetic estimation of the apparent activation Gibbs free energy (G_a) of methanol formation and the Reverse Water-Gas Shift (RWGS) as a function of the number of Pd atoms present in the Pd-doped In_2O_3(111) system. These estimated values were taken from Reference.[10]

System	Exposed Pd atoms	$G_{a,MeOH}$ / kJ mol^{-1}	$G_{a,RWGS}$ / kJ mol^{-1}
In_2O_3(111)	–	137	127
CP_a	0	51	245
CP_b	1	81	333
CP_c	2	133	159
CP_d	3	405	247
DI_a	1	87	154
DI_b	3	353	148
Pd(111)	All	230	166

Table S4: Mechanism of CO_2 hydrogenation to methanol on Pd(111). "*" denotes an adsorption site on the catalyst surface.

Label	Reaction	Label	Reaction
1	$H_{2(g)} + 2* \rightarrow 2H^*$	9	$CH_3O^* + H^* \rightarrow CH_3OH^* + *$
2	$CO_{2(g)} + * \rightarrow CO_2\ ^*$	10	$CO^* + H^* \rightarrow COH^* + *$
3	$CO_2^* + H^* \rightarrow COOH^* + *$	11	$COH^* + H^* \rightarrow CHOH^* + *$
4	$COOH^* + * \rightarrow CO^* + OH^*$	12	$CHOH^* + H^* \rightarrow CH_2OH^* + *$
5	$H^*+OH^* \rightarrow H_2O^* + *$	13	$CH_2OH^* + H^* \rightarrow CH_3OH^* + *$
6	$CO^* + H^* \rightarrow CHO^* + *$	14	$H_2O^* \rightarrow H_2O_{(g)} + *$
7	$CHO^* + H^* \rightarrow CH_2O^* + *$	15	$CO^* \rightarrow CO_{(g)} + *$
8	$CH_2O^* + H^* \rightarrow CH_3O^* + *$	16	$CH_3OH^* \rightarrow CH_3OH_{(g)} + *$

Table S5: Mechanism of CO_2 hydrogenation to methanol on In_2O_3.[12] "*" denotes an adsorption site on the catalyst surface.

Label	Reaction
1	$H_{2(g)} + 2^* \rightarrow 2H^*$
2	$CO_{2(g)} + {}^* \rightarrow CO_2{}^*$
3	$CO_2^* + H^* \rightarrow CHO_2^* + {}^*$
4	$CHO_2^* + H^* \rightarrow CHO_2H^* + {}^*$
5	$CHO_2H^* + H^* \rightarrow CH_2O_2H^* + {}^*$
6	$CH_2O_2H^* + {}^* \rightarrow CH_2O + OH^*$
7	$OH^* + H^* \rightarrow H_2O^* + {}^*$
8	$CH_2O^* + H^* \rightarrow CH_3O^* + {}^*$
9	$CH_3O^* + H^* \rightarrow CH_3OH^* + {}^*$
10	$CHO_2^* + H^* \rightarrow CH_2O_2^* + {}^*$
11	$CH_2O_2 + H^* \rightarrow CH_2O_2H^* + {}^*$
12	$CH_2O_2H^* + H^* \rightarrow CH_2(OH)_2^* + {}^*$
13	$CH_2(OH)_2^* + H^* \rightarrow CH_2OH^* + H_2O^*$
14	$CO_2^* + H^* \rightarrow COOH^* + {}^*$
15	$COOH^* + H^* \rightarrow CO^* + H_2O^*$
16	$H_2O^* \rightarrow H_2O_{(g)} + {}^*$
17	$CO^* \rightarrow CO_{(g)} + {}^*$
18	$CH_3OH^* \rightarrow CH_3OH_{(g)} + {}^*$

Table S6: Labels of reaction species (adsorbed and in gas phase) identified by AutoProfLib, together with their explanations.

Label	State	Label	State
i15	$*$	i25	$H_2COOH^*+3H^*$
i16	$2H^*$	i26	$OCH_2^*+2H^*+H_2O^*$
i17	CO_2^*	i27	$OCH_2^*+2H^*$
i18	$CO_2^*+2H^*$	i28	$H_3CO^*+H^*$
i20	$HCO_2^*+H^*$	i29	H_3COH^*
i21	$HCO_2^*+3H^*$	i35	H_2O^*
i22	$H_2CO_2^*+2H^*$	i38	$COOH^*+OH^*$
i23	$HCOOH^*+2H^*$	i39	$CO^* + H_2O^*$
i24	$H_2COOH^*+H^*$	CO2	$CO_{2(g)}$
H2	$H_{2(g)}$	Methanol	$CH_3OH_{(g)}$
CO	$CO_{(g)}$	H2O	$H_2O_{(g)}$

Table S7: Direct and reverse barriers for the COOH and the CHOO intermediates formation, $G_{a,COOH}$ and $G_{a,CHOO}$ respectively. Barriers obtained from the data provided in Ref.[10]

	$G_{a,COOH}$ / eV		$G_{a,CHOO}$ / eV	
Model	direct	reverse	direct	reverse
CP_a	1.01	1.17	0.00	0.36
CP_b	0.24	0.44	0.00	0.10
CP_c	0.90	1.01	0.15	0.00
CP_d	0.42	0.04	1.21	0.00
DI_a	0.67	0.76	0.15	1.25
DI_b	0.79	0.62	0.76	0.00
In_2O_3	0.43	0.46	0.44	0.00
Pd	1.28	0.20	–	–

Table S8: Degree of Rate Control (DRC) analysis for the CP_a model in CO_2 hydrogenation to methanol and reverse water-gas shift (RWGS). The intermediates i are labeled corresponding to **Table S6**.

Step	DRC_{MeOH}	DRC_{RWGS}	Reaction
R1	0.00	0.00	$H_{2(g)}$ + i15 → i16
R2	0.00	0.00	$CO_{2(g)}$ + i15 → i17
R3	0.00	0.00	$CO_{2(g)}$ + i16 → i18
R4	0.00	0.00	$H_{2(g)}$ + i17 → i18
R5	0.00	0.00	i18 → i20
R6	0.00	0.00	$H_{2(g)}$ + i20 → i21
R7	0.00	0.00	i21 → i22
R8	0.00	0.00	i22 → i24
R9	0.00	0.00	i21 → i23
R10	0.00	0.00	i23 → i24
R11	0.06	0.00	$H_{2(g)}$ + i24 → i25
R12	0.42	0.00	i25 → i26
R13	0.42	0.00	i26 → i27 + $H_2O_{(g)}$
R14	0.00	0.00	i27 → i28
R15	0.10	0.00	i28 → i29
R16	0.00	0.00	i29 → i15 + $CH_3OH_{(g)}$
R17	0.00	0.92	i18 → i38
R18	0.00	0.08	i38 → i39
R19	0.00	0.00	i39 → i35 + $CO_{(g)}$
R20	0.00	0.00	i35 → i15 + $H_2O_{(g)}$
Total	1.00	1.00	

References

(1) Grimme, S. Supramolecular binding thermodynamics by dispersion-corrected density functional theory. *Chem. Eur. J.* **2012**, *18*, 9955–9964.

(2) Hjorth Larsen, A. et al. The atomic simulation environment—a Python library for working with atoms. *J. Phys. Condens. Matter* **2017**, *29*, 273002.

(3) McQuarrie, D.; Simon, J. *Molecular Thermodynamics*; University Science Books, 1999.

(4) Pablo-García, S.; Sabadell-Rendón, A.; Saadun, A. J.; Morandi, S.; Pérez-Ramírez, J.; López, N. Generalizing Performance Equations in Heterogeneous Catalysis from Hybrid Data and Statistical Learning. *ACS Catal.* **2022**, *12*, 1581–1594.

(5) Wolcott, C. A.; Medford, A. J.; Studt, F.; Campbell, C. T. Degree of Rate Control Approach to Computational Catalyst Screening. *J. Catal* **2015**, *330*, 197–207.

(6) Filot, I. A. W. *Introduction to microkinetic modeling*, 1st ed.; Technische Universiteit Eindhoven: Eindhoven, 2018.

(7) Maestri, M.; Cuoci, A. Coupling CFD with detailed microkinetic modeling in heterogeneous catalysis. *Chem. Eng. Sci.* **2013**, *96*, 106–117.

(8) Maffei, T.; Gentile, G.; Rebughini, S.; Bracconi, M.; Manelli, F.; Lipp, S.; Cuoci, A.; Maestri, M. A multiregion operator-splitting CFD approach for coupling microkinetic modeling with internal porous transport in heterogeneous catalytic reactors. *Chem. Eng. J.* **2016**, *283*, 1392–1404.

(9) Weller, H. G.; Tabor, G.; Jasak, H.; Fureby, C. A tensorial approach to computational continuum mechanics using object-oriented techniques. *Comput. phys.* **1998**, *12*, 620–631.

(10) Frei, M. S.; Mondelli, C.; García-Muelas, R.; Kley, K. S.; Puértolas, B. n.; López, N.; Safonova, O. V.; Stewart, J. A.; Curulla Ferré, D.; Pérez-Ramírez, J. Atomic-scale engineering of indium oxide promotion by palladium for methanol production via CO2 hydrogenation. *Nat. Commun.* **2019**, *10*, 3377.

(11) 3rd Millenium Database. https://publications.anl.gov/anlpubs/2005/07/53802.pdf, Accessed: 2021-11-15.

(12) Frei, M. S.; Capdevila-Cortada, M.; García-Muelas, R.; Mondelli, C.; López, N.; Stewart, J. A.; Ferré, D. C.; Perez-Ramirez, J. Mechanism and microkinetics of methanol synthesis via CO2 hydrogenation on indium oxide. *J. Catal.* **2018**, *361*, 313–321.
